# Candida Infections in Marine Mammals: Epidemiology, Antifungal Resistance, and One Health Implications

**DOI:** 10.3390/ani16071060

**Published:** 2026-03-31

**Authors:** Michelyne Haroun, Christophe Tratrat, Muhammad Munir, Ouda Nasser Aldakhilallah, Sahar Mohamed Ibrahim, Athina Geronikaki

**Affiliations:** 1 Fish Resources Research Center, King Faisal University, Al-Ahsa 31982, Saudi Arabia; oaldakhilalla@kfu.edu.sa; 2 Department of Pharmaceutical Sciences, College of Clinical Pharmacy, King Faisal University, Al-Ahsa 31982, Saudi Arabia; ctratrat@kfu.edu.sa; 3 Date Palm Research Center of Excellence, King Faisal University, Al-Ahsa 31982, Saudi Arabia; 4 Avian Research Center, King Faisal University, Al-Ahsa 31982, Saudi Arabia; 5 Department of Pharmacy Practice, College of Clinical Pharmacy, King Faisal University, Al-Ahsa 31982, Saudi Arabia; smibrahim@kfu.edu.sa; 6 Department of Pharmaceutical Chemistry, School of Pharmacy, Aristotle University of Thessaloniki, 54124 Thessaloniki, Greece; geronik@pharm.auth.gr

**Keywords:** antifungal resistance, *Candida albicans*, conservation, diagnosis, disease prevention, marine mammals, non-*albicans Candida*, One Health, treatment, wildlife infection

## Abstract

*Candida albicans* is currently considered one of the most significant fungal pathogens in cetaceans and pinnipeds and the spread of antifungal-resistant strains pose significant threats to animal health and One Health concerns. Although *C. albicans* is the most commonly detected species, non-*albicans Candida* (NAC) species, including *C. tropicalis*, *C. parapsilosis* and *Nakaseomyces glabratus* and the multidrug-resistant *C. auris*, have been recognized in captive dolphins. This review examines the clinical patterns observed in marine mammal taxa: cetaceans are most commonly vulnerable to respiratory and disseminated mycoses owing to their distinct anatomical characteristics, whereas mucocutaneous infections are the common manifestation in pinnipeds. Localized mucocutaneous infections may progress to fatal systemic disease, with mortality rates approaching 100% in severe cases, despite therapeutic treatment. The most important predisposing factors are immunosuppression, long-term antibiotic treatment, environmental stress factors, and the deterioration of water quality. Diagnostic methods are based on cytology, histopathology, culture, and molecular methods, and treatment is mostly composed of systemic azole antifungals although with high levels of therapeutic failure. Recent results showed that there are high levels of azole resistance in the isolates of marine mammals that had no history of exposure to antifungal agents, which points to the role of aquatic environments as sources of resistance genes. The lack of knowledge remains particularly evident in species-specific pharmacokinetics and the development of evidence-based treatment guidelines. These infections also have broader implications for ecosystem health surveillance and the protection of endangered marine mammal populations. The current review highlights the One Health approach with marine mammals being at the core of ocean health surveillance and identifies the potential for zoonotic transmission.

## Simple Summary

This scientific review of available literature recognizes *Candida* infections in cetaceans and pinnipeds as an emerging health concern, with One Health implications. Although *Candida albicans* is still the most commonly isolated species among marine mammals, other species, including *C. tropicalis*, *C. parapsilosis*, and *Nakaseomyces glabratus*, along with multidrug-resistant *C. auris*, have been detected in captive dolphins, indicating a notable shift in species distribution within the studied facilities that warrants further monitoring. Notably, resistance rates of up to 100% have been reported in individual facilities, and resistant isolates have been obtained in animals unexposed to antifungal treatments, suggesting that aquatic environments may serve as reservoirs of resistance determinants, although broader datasets are needed to confirm these observations. Clinical manifestations differ markedly between host groups: while cetaceans are mostly affected by respiratory and systemic mycoses that start at the blowhole and result in deadly outcomes, pinnipeds more frequently present with mucocutaneous infections. There are still notable gaps in the knowledge about species-specific antifungal pharmacokinetics, standardized diagnostic guidelines and evidence-based treatment methods. Finally, candidiasis in marine mammals should not be considered solely a veterinary concern but as an indicator of the environmental health with possible negative consequences for the marine ecosystem.

## 1. Introduction

New and re-emerging infectious diseases pose a growing threat to both human and wildlife health, with marine ecosystems increasingly recognized as vulnerable environments for pathogen emergence [1,2]. Marine mammals such as cetaceans and pinnipeds are important indicators of ocean ecosystem health and are subject to many anthropogenic and environmental hazards [3]. There has been a heightened incidence and severity of disease in marine species especially due to fungal-induced diseases in recent years [4]. This trend is partly driven by climate change, which promotes the selection of thermotolerant fungal strains, potentially narrowing the gap between environmental temperatures and mammalian body temperature [5]. The ecological and economic impact of these diseases can be overwhelming, as they can affect the health of marine organisms [3,6].

Among marine organisms, fungal infections have become an increasingly important health concern, with *Candida* species considered significant opportunistic pathogens capable of inducing severe morbidity and mortality [7]. In these species, candidiasis often presents as debilitating lesions in the oral cavity and digestive tract, such as glossitis and stomatitis, which directly impair the animal’s ability to feed and maintain proper nutrition [7]. In many cases, cutaneous candidiasis manifests as persistent, multifocal skin plaques or ulcers that compromise the dermal barrier, leading to secondary bacterial infections and chronic stress in affected individuals [3]. Furthermore, the progression to localized or systemic respiratory infections can be fatal for diving mammals, as compromised lung function severely limits their foraging capacity and survival in the wild [3]. The first detailed reports of candidiasis in marine mammals appeared in the early 1980s, including the occurrence of widespread *Candida albicans* infections in pinnipeds and captive cetaceans [8,9]. Since these pioneering observations, knowledge about candidiasis in marine mammals has increased tremendously, providing complex interactions between fungal virulence factors, host immune responses, environmental factors, and anthropogenic factors [3,8,9]. Regardless of these advances, the particular molecular mechanisms of *Candida* pathogenesis in marine hosts, which include contact-sensing and biofilm-mediated resistance, are not adequately described in comparison with the human clinical paradigm [10]. There are several factors that influence the clinical significance of Candida infections in marine mammals. First, these infections may lead to severe morbidity and even mortality, particularly in individuals experiencing some form of stress, or with compromised immune systems [8,11]. In addition to causing direct mortality, chronic fungal infections may cause reproductive failure and reduced maternal care, which further reduces the populations of declining marine mammals already under stress due to habitat loss [12]. Diversified physiological responses in the form of diving-related oxidative stress of marine mammals could also contribute to these immune–pathogen interactions in a manner that has yet to be comprehensively understood [13].

Second, the appearance of antifungal resistance in *Candida* marine isolates is concerning, as it may parallel trends in human healthcare and pose clinical challenges [14,15]. Third, zoonotic and reverse zoonotic transmission is a potential health threat to marine mammal workers. Consequently, marine mammals as sentinel species can detect broader environmental health trends, including the presence of harmful and resistant fungi in the marine environment [3,12].

The review gives an overview of the existing data on *Candida* infections in cetaceans and pinnipeds evaluating epidemiology, clinical manifestations, pathogenesis, diagnosis, treatment and the larger impacts of marine mammal health and conservation through the One Health prism [16,17]. The recent *Candida auris* identified in a captive dolphin has been a breakthrough discovery in the marine mammal mycology, as the multidrug-resistant pathogen poses tremendous challenges for treatment and infection control [18]. This finding, together with the reports of 100% azole resistance of *Candida* strains in dolphins that have never been exposed to antifungals, illustrates the need to increase fungal surveillance in marine mammals [19].

For this narrative review, relevant literature was identified through searches of PubMed, Web of Science, Scopus, and Google Scholar databases using combinations of terms including ‘*Candida*’, ‘marine mammal’, ‘cetacean’, ‘pinniped’, ‘antifungal resistance’, and ‘One Health’. The search covered publications from 1975 to March 2026, with no language restrictions. Inclusion criteria comprised original research articles, case reports, case series, and reviews reporting *Candida* species isolation, infection, colonization, antifungal susceptibility, or treatment in cetaceans or pinnipeds.

## 2. Candida Species Distribution in Marine Mammals

Several *Candida* species have been identified in marine mammal populations with different levels of pathogenicity and clinical significance [20]. The distribution of these species varies among cetaceans and pinnipeds depending on geographic location, environmental conditions, and whether animals are captive or free-ranging. Studies of wild bottlenose dolphins in the Gulf of Mexico and the Atlantic Ocean have documented varying levels of *Candida* colonization [21], while investigations of captive dolphins in Japanese aquaria have revealed high prevalence rates [22] (Table 1).

### 2.1. Candida albicans

*Candida albicans* remains the most frequently isolated and clinically significant species in marine mammals according to Reidarson et al. [7]. This species has been documented in bottlenose dolphins, beluga whales, harbor porpoises, pilot whales and various pinniped species, including harbor seals, California sea lions, and walruses [8,24,34]. In these species, the pathogen is frequently recovered from the blowhole, gastric fluid, and mucocutaneous lesions. *C. albicans* possesses extensive virulence attributes including dimorphic switching, adhesin expression, biofilm formation, and secretion of hydrolytic enzymes that facilitate tissue invasion and immune evasion [35]. Studies of wild bottlenose dolphin populations in Sarasota Bay, Florida, by Buck et al., revealed that 7.0% of examined individuals harbored *C. albicans* in their mucosal microbiota, with *C. tropicalis* occurring in 14.3% of animals, indicating that colonization is common even in healthy free-ranging animals [21]. The transition from colonization to disease depends on complex host-pathogen-environment interactions [7].

### 2.2. Non-Albicans Candida Species

Other non-*albicans Candida* species identified in marine mammals often exhibit intrinsic or acquired resistance to most commonly used antifungal agents [14]. *Candida tropicalis*, which has been isolated in many cetacean species, including the bottlenose dolphins, has emerged as one of the most significant *Candida* species due to its correlation with azole resistance and the invasive disease [14,19,22]. The species is especially common in the oceanic environment in tropical and subtropical areas and is potentially more aggressive than *C. albicans* in the presence of immunocompromised hosts [10].

*Candida parapsilosis* represents another clinically important species, documented in dusky dolphins [24]. In these dolphins, isolates were obtained from the blowhole, stomach fluid, mouth, anus, and feces. This species is ubiquitous in environmental samples and can be isolated from soil and aquatic environments. *C. parapsilosis* has been associated with biofilm formation on medical devices in human medicine [36]. *Nakaseomyces glabratus* (formerly *Candida glabrata*) has been isolated from the respiratory tract and gastric fluid of bottlenose dolphins in Japanese aquaria, with some of the isolates being resistant to the azole antifungals [14]. This species has a lower susceptibility to fluconazole and is prone to develop resistance during treatment [7].

Other non-*albicans Candida* have been reported in other marine mammals. *Candida zeylanoides* has been isolated from a beached southern right whale neonate in South Africa [32]. A number of *Candida* species, including *Candida ciferrii*, *Candida lambica*, *Meyerozyma guilliermondii* (formerly *Candida guilliermondii*) as well as *Debaryomyces hansenii* (formerly *Candida famata*) have been reported in beluga whales and other cetaceans, but epidemiological information is still scarce [20,21,32,37].

### 2.3. Geographic and Environmental Distribution

The geographical distribution and the environmental conditions are the factors that determine the prevalence of *Candida* species among marine mammals [10]. High prevalence of azole-resistant *Candida* species has been reported in Japanese aquaria studies, possibly caused by the water treatment procedure or to local environmental conditions [10]. The presence of fungi in various locations can be affected by the environmental factors like salinity, pH of the soils, nitrogen content, and climate [38]. *Candida* species diversity seems to be more abundant in the tropical and subtropical waters, with *C. tropicalis* specifically prevalent in warmer waters [39]. Human-related *Candida* species are acquired by wild cetaceans, thus indicating coastal waters contaminated with anthropogenic-related yeasts [10]. *Candida albicans* has been suggested as an indicator of fecal water contamination [38]. *Candida* spp. prevalence was reported at 70% in dolphins, 90% in pool water, and 29% among staff members, suggesting extensive contamination of the environment by *Candida* spp. and possible inter-specific transmission [25]. Free-ranging bottlenose dolphin populations in the Gulf of Mexico and Atlantic Ocean have exhibited different levels of *Candida* colonization, which provides a baseline of data on environmental transmission studies [21]. Among others, the first outbreak of the *Candida auris* colonization in a captive dolphin in the Dominican Republic was reported by Ferrara et al. [18]. *C. auris* being detected in cetaceans is a major issue concerning the existence of environmental reservoirs and the possibility of inter-species transmission of this multidrug-resistant pathogen [18].

It is important to note that the current body of literature on Candida infections in marine mammals is predominantly composed of individual case reports and small-scale surveillance studies from a limited number of facilities. The geographic distribution of published data is heavily biased toward Japan [14,15,22,25], the USA [8,9,21], and Italy [19], reflecting the research activity and diagnostic capacity of specific institutions rather than the true global epidemiology of marine mammal candidiasis. The majority of reports originate from captive populations, where intensive veterinary monitoring facilitates detection, while data from free-ranging populations remain scarce, likely underestimating the true prevalence of infection in wild marine mammals. Furthermore, a publication bias toward severe and fatal cases may overrepresent the clinical significance of candidiasis, while subclinical or self-resolving infections are less frequently reported. These biases should be considered when interpreting the species distribution, prevalence rates, and resistance patterns described in this review. Multi-center, standardized epidemiological studies across diverse geographic regions and both captive and wild populations are needed to establish a more comprehensive understanding of Candida epidemiology in marine mammals.

## 3. Clinical Manifestations

There are various clinical manifestations of candidiasis in marine mammals, starting with a superficial infection of the mucosa and progressing to a deadly systemic disease [10]. Clinical spectrum comprises respiratory, dermal, gastrointestinal, and systemic affliction with increasing cases of antifungal resistance, making diagnosis and treatment difficult [7]. Clinical presentation differs with the involvement of different *Candida* species, as well as host species, immune status, and the presence of predisposing factors [7,8].

### 3.1. Clinical Manifestations and Pathological Findings of Candidiasis in Cetaceans

**Figure 1:**
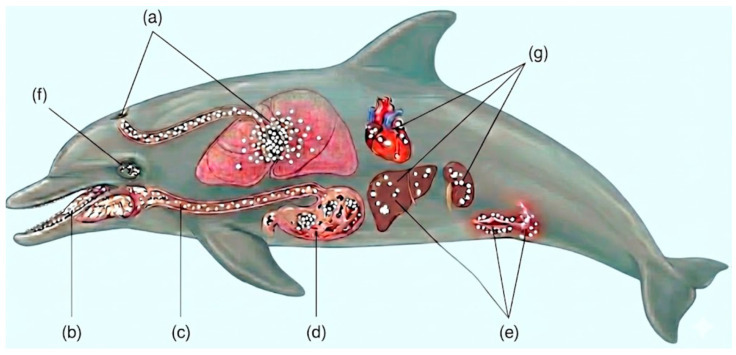
Schematic illustration of potential clinical manifestations of candidiasis in dolphins (cetaceans). White dots indicate sites of infection. (a) Respiratory tract (blowhole and lungs); (b) oral cavity (tongue, buccal mucosa, and pharynx); (c) esophagus; (d) gastrointestinal tract (stomach and intestines); (e) cutaneous lesions around body orifices (genital slit, anus); (f) ocular infection (keratomycosis); (g) systemic dissemination sites (heart, liver, kidney, spleen, lymph nodes, and brain).

Reported cases of candidiasis in captive cetaceans have established the basis of knowledge in disease trends of these species [8]. A thorough literature research on mycotic diseases in marine mammals, such as candidiasis in marine mammals, gives vital clinical advice to veterinarians treating cetacean populations [7]. The anatomical distribution and diversity of candidiasis manifestations in cetaceans are illustrated in Figure 1.

#### 3.1.1. Respiratory Candidiasis

Candidiasis pulmonary infection is one of the most significant clinical manifestations of infection in cetaceans [10]. The unique anatomy of cetaceans, which lack nasal turbinates, may permit fungal pathogens such as *Candida* yeasts to find their way to the lungs with greater ease, predisposing these animals to respiratory mycoses [7]. The blowhole and other related breathing passages provide a conducive environment for the growth of fungi. Affected animals may present with discharge from the blowhole [14]. Clinical manifestations recorded among infected dolphins entail foul breath, fever, neutrophilia, leukocytosis, increased fibrinogen, and increased erythrocyte sedimentation rate [14]. In advanced cases of respiratory tract disease in dolphins, clinical signs of malaise, anorexia, increased respiratory rate, coughing and foul smelling breath may be demonstrated [24,40]. Investigations of blowhole samples in captive dolphins have also shown recurrently high prevalence of *Candida* colonization, with implications for respiratory health [14,22]. In Japanese aquaria, several bottlenose dolphin cases involving *Candida* colonization of respiratory tract were reported, with some animals harboring azole-resistant strains [14,15,22]. In the Okinawa Churaumi Aquarium, *C. albicans* and *C. tropicalis* were isolated from expired breaths of 70% of captive dolphins [22]. Moreover, in a study conducted at the Enoshima Aquarium, all eight *C. albicans* strains isolated from dolphin expired breath were resistant to fluconazole, itraconazole, and voriconazole, but were susceptible to amphotericin B and micafungin [15]. Respiratory candidiasis can be localized or act as a transmission route, particularly in immunocompromised animals [10]. The early detection through the routine sampling of blowholes has gained significance as a diagnostic method in controlled cetacean populations [10]. Marques et al. designed a practical in-house respiratory fungal culture protocol in the case of the bottlenose dolphins at Zoomarine, Portugal, by implementing quarterly screening of the sputum samples as part of the preventive medicine program [41]. The protocol involved Sabouraud Dextrose Chloramphenicol Agar, as well as CHROMagar *Candida* Plus, which can differentiate between *C. albicans*, *N. glabratus* (*C. glabrata*), *C. tropicalis*, *P. kudriavzevii* (*C. krusei*), and especially *C. auris*. The treatment resulted in the identification of respiratory infections caused by *Cunninghamella bertholletiae*, *Aspergillus fumigatus*, *A. flavus*, *C. albicans*, and *N. glabratus* (*C. glabrata*) over three years of surveillance, demonstrating the usefulness of routine mycological surveillance in captive cetaceans [41].

#### 3.1.2. Oral and Oropharyngeal Candidiasis

In cetaceans, oral candidiasis is characterized by white raised lesions that turn into red circular lesions in the mouth (particularly tongue) and the esophagus [8]. The lesions involve masses of septate, branching hyphae within the stratified squamous epithelium along the edges of large areas of ulceration [8]. Affected animals can show anorexia and apparent gastric distress, manifested by head shaking, discharging air through the blowhole while under water, vomiting, and reluctance to swallow food that has been mouthed [8]. Dusky dolphins (*Lagenorhynchus obscurus*) with oral and cutaneous candidiasis have been reported to present oral thrush with a white coating on the tongue and ulceration of the tongue revealed after eradication of *C. albicans* [24].

#### 3.1.3. Cutaneous Candidiasis

Cutaneous candidiasis in cetaceans typically presents as chronic lesions affecting areas around body orifices [8,42]. The lesions are erythematous, erosive, with white pseudomembranous plaques analogous to those encountered in oral disease [8,11]. The effects of chronic infections might be the thickening of the skin, hyperpigmentation, and scarring [43]. Affected sites commonly include areas around the genital slit, the anus, and other body orifices, where moisture accumulation predisposes to fungal colonization [8]. *Candida* species such as *C. ciferrii* and *C. lambica* were also isolated from the blowhole (*C. lambica*) and from anal swabs (*C. ciferrii*) in beluga whales (*Delphinapterus leucas*), but their pathogenicity, as opposed to opportunistic colonization, needs to be closely interpreted [20]. Chronic cutaneous candidiasis was recognized in captive performing bottlenose dolphins, wherein persistent skin lesions along body orifices could not respond to standard antimicrobial therapy but improved with immunopotentiating treatment with levamisole phosphate [11]. Cetacean cutaneous candidiasis may occur as a localized infection or as part of disseminated systemic disease involving internal organs [8]. In captive cetaceans, cutaneous candidiasis is strongly affected by the environmental factors. High risk of cutaneous fungal infections has been linked to excessive chlorination of pool water, sub-optimal water quality, and high water temperatures [33].

#### 3.1.4. Gastrointestinal Candidiasis

Candidiasis in cetaceans is often reported to involve the esophagus and the stomach [8]. The ulcerative lesions that are characterized by pseudomembranous plaques spreading out of the oral cavity through the esophagus to the stomach are often identified by using endoscopic and post-mortem examination [7]. The gastrointestinal disorder takes the form of gastric or esophageal distress; the ailing dolphins often appear with anorexia, reluctance to swallow mouthed food, head shaking, vomiting, and retching [8,20]. Clinical signs include anorexia, and the animal may stop eating abruptly, or it may mouth food as if swallowing were painful [8]. Candidiasis of the intestines, rarely diagnosed during life, has been reported at the necropsy [31]. An example of such an incident was chronic intestinal candidiasis produced by *Pichia kudriavzevii* (formerly *Candida krusei*), an azole-intrinsically resistant species in an aged captive Pacific white-sided dolphin, which resulted in intestinal torsion and demise [31]. This case accentuated the acuteness of gastrointestinal candidiasis and the problems associated with intrinsically resistant *Candida* species. Biancani et al. described a case of gastrointestinal candidiasis in a bottlenose dolphin, revealing clinical signs of gastrointestinal discomfort, anorexia, and abnormal feces that tested positive for yeast cells after performing cytology examination [19]. Combination therapy of fluconazole and terbinafine was administered and successfully treated the animal, with total resolution, as evidenced by two negative fecal cultures biweekly [19].

#### 3.1.5. Ocular Candidiasis

*Candida albicans* has been reported to cause keratomycosis in bottlenose dolphins, in the form of corneal ulceration due to secondary fungal infection [23]. The infected animals exhibited bilateral diffuse corneal opacities, blepharospasm (spasm of eyelids), epiphora (excessive tearing), and photophobia (sensitivity to light) [23]. The infection is normally produced by corneal trauma, due to foreign bodies, aggressive interaction, or irritation caused by chemically treated pool water [23]. Diagnosis requires corneal cytology, fungal culture and, if necessary, corneal biopsy to ensure fungal invasion and not superficial colonization [23]. The therapy requires intensive topical and systemic antifungal therapy, such as frequent topical antifungal therapy (natamycin, voriconazole), systemic antifungals (fluconazole, itraconazole), and supportive care to maintain vision and avoid the development of endophthalmitis [23]. New interventions, such as adipose-derived stem cells, have been discovered to have potential in recalcitrant cases [23]. Successful management required months of therapy with close monitoring [23].

#### 3.1.6. Systemic and Disseminated Candidiasis

Disseminated candidiasis is the most severe form of infection in cetaceans, with a high mortality rate [8]. The infection may arise from extension of mucocutaneous disease or through hematogenous dissemination from the gastrointestinal tract [44]. Clinical manifestations are usually non-specific and might include lethargy, changes in behavior, chronic fever resistant to antibiotic treatment, cutaneous lesions spread over body surfaces, multi-organ dysfunction, anorexia, weight loss, and progressive deterioration despite supportive care [7]. In disseminated candidiasis, post-mortem examination reveals fungal granulomas in various body parts, such as lungs, heart, kidney, liver, spleen, lymph node, and the brain [8]. Yeast and pseudohyphal forms of tissue invasion are shown by histopathology, accompanied by granulomatous inflammation and tissue necrosis [8]. Disseminated candidiasis is usually lethal in cetaceans, despite the intensive therapy [10].

### 3.2. Clinical Manifestations and Pathological Findings in Pinnipeds

**Figure 2:**
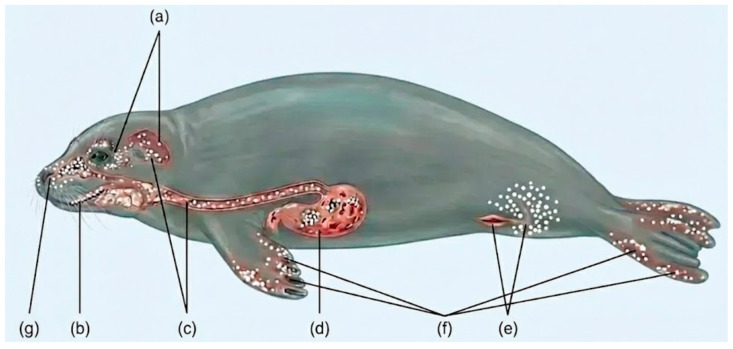
Schematic illustration of potential clinical manifestations of candidiasis in pinnipeds. White dots indicate sites of infection. (a) Periocular candidiasis: alopecia and inflammation around the eyes; (b) oral candidiasis: commissures of the mouth; (c) esophageal candidiasis *; (d) gastric candidiasis *; (e) mucocutaneous candidiasis: anogenital region (anus, vagina); (f) cutaneous candidiasis: flippers (nail beds); white plaques, alopecia; (g) respiratory candidiasis: nares (nasal discharge). * Esophageal and gastric candidiasis have not been specifically documented as case reports in pinnipeds but have been reported as possible manifestations in marine mammals [7,10].

Pinnipeds are susceptible to candidiasis, which is mostly associated with mucocutaneous junctions and accompanied by dermatitis of the body orifices. Dunn et al. [9] were the first researchers to thoroughly examine the prevalence of candidiasis infection in captive pinnipeds and reported the disease in five species: gray seal (*Halichoerus grypus*), harbor seal (*Phoca vitulina*), northern fur seal (*Callorhinus ursinus*), California sea lion (*Zalophus californianus*), and northern elephant seal (*Mirounga angustirostris*). Pinnipeds tend to express mucocutaneous rather than respiratory or systemic disease, as compared to cetaceans. A recent survey of marine mammals stranded along the German coast revealed 32 Candida cases across three species, including 18 in harbor seals and five in gray seals, suggesting that candidiasis may be more prevalent in wild pinnipeds than previously recognized [7,10]. An anatomical display of candidiasis manifestations in pinnipeds [9] is shown in Figure 2.

#### 3.2.1. Respiratory Candidiasis

Respiratory involvement in pinniped candidiasis has been documented primarily as purulent nasal discharge associated with *Candida albicans* infection [9]. Unlike cetaceans, in which pulmonary candidiasis is a major clinical manifestation, lower respiratory tract *Candida* infection has not been well documented in pinnipeds. However, mycotic pneumonia caused by other fungal agents, such as *Aspergillus* and *Coccidioides*, has been reported in California sea lions and harbor seals [7].

#### 3.2.2. Oral and Oropharyngeal Candidiasis

Oral candidiasis in pinnipeds manifests as inflammation at the mucocutaneous junctions, particularly at the commissures of the mouth [9]. In phocids, oral lesions frequently occur concurrently with periocular and anogenital involvement as part of a broader mucocutaneous disease pattern [9].

#### 3.2.3. Cutaneous Candidiasis

Candidiasis in pinnipeds, especially phocids (true seals), mostly affects the mucocutaneous junctions [9,33]. Characteristic lesions are formed at the commissures of the mouth, in the periocular areas, and around the nares, anus, and vagina [9]. These areas present with white plaques, alopecia, and occasionally ulceration with purulent discharge [9]. Vaginitis is common in female pinnipeds, manifesting as vulvar discharge and inflammation [9]. In gray seals (*Halichoerus grypus*) and harbor seals (*Phoca vitulina*), dermatitis has been reported to affect the flippers, especially around the nail beds [9,33]. Various species of affected pinnipeds were reported, such as harbor seals, California sea lions (*Zalophus californianus*), northern fur seals (*Callorhinus ursinus*), gray seals, and northern elephant seals (*Mirounga angustirostris*) [9,33]. Fungal dermatitis in captive pinnipeds has also been associated with *Yarrowia lipolytica* (previously known as *Candida lipolytica*) [33]. Pollock et al. reported the isolation of *Y. lipolytica*, along with *Trichophyton mentagrophytes* and *Malassezia* spp., from two gray seals and four harbor seals [33]. Affected animals had erythematous thickened, alopecic lesions on the face (the muzzle and periorbital region) and flippers, especially around the nail beds, with additional lesions on the trunk and tail. A California sea lion housed in the same facility also developed ulcerated flipper lesions from, which *Malassezia* spp. and *Candida zeylanoides* were isolated [33]. Several environmental conditions, such as excessive chlorination of pool water and elevated water temperatures, appear to favor the occurrence of fungal dermatitis [33,45].

#### 3.2.4. Gastrointestinal Candidiasis

Gastrointestinal candidiasis has not been specifically documented as individual case reports in pinnipeds. However, diminished appetite has been reported as a clinical sign in pinnipeds with candidiasis, which may suggest gastrointestinal involvement [9]. Reidarson et al. reported that clinicians have observed a rise in gastrointestinal candidiasis in marine mammals, including pinnipeds [7]. A survey of stranded marine mammals along the German coast identified *Candida* in 18 harbor seals (*Phoca vitulina*) and five gray seals (*Halichoerus grypus*), although the specific anatomical sites of isolation were not detailed [10]. The gastrointestinal tract serves as a reservoir for *Candida* species and may become a site of invasive disease, particularly when normal bacterial flora is disrupted by antibiotic treatment [7]. *Candida* spp. have been detected in stomach samples of marine mammals; however, the distinction between colonization and true infection requires cytological evidence of local tissue invasion, such as the presence of budding yeasts, hyphae, or pseudohyphae, along with inflammatory cells [7].

#### 3.2.5. Ocular Candidiasis

Only periocular candidiasis, presenting as alopecia and inflammation around the eyes, has been documented in pinnipeds [9]. Ocular surface disease is common in captive pinnipeds, with progressive keratitis affecting up to 43% of animals [46,47]. While primary *Candida* keratitis has not been specifically reported, secondary fungal infections can complicate progressive otariid keratopathy [46]. *Candida*-associated intraocular infection (endophthalmitis) has not been documented in pinnipeds, in contrast to cetaceans, in which keratomycosis due to *C. albicans* has been reported [23].

#### 3.2.6. Systemic and Disseminated Candidiasis

Disseminated candidiasis specifically caused by *Candida* species has not been well documented in pinnipeds. However, systemic mycoses caused by other fungal pathogens have been reported, including *Coccidioides immitis* in California sea lions and harbor seals [48,49], *Cryptococcus* species [50], and *Cystofilobasidiales* [51]. Sweeney et al. provided early documentation of systemic mycoses in marine mammals, noting that immunocompromised animals are predisposed to opportunistic fungal infections, including *Candida* [7]. The relative rarity of disseminated candidiasis in pinnipeds compared to cetaceans may reflect differences in disease susceptibility or reporting bias [7,10].

### 3.3. Comparison Between Cetaceans and Pinnipeds

Clinical signs of candidiasis show significant dissimilarities between cetaceans and pinnipeds, reflecting differences in their anatomy, physiology, and environmental niches. Cetaceans are more commonly predisposed to respiratory and systemic infections. This susceptibility is potentially related to their obligatory marine environment, the specific anatomy of their respiratory tract (the blowhole), and challenges in regulating water quality in captive facilities, which position the blowhole and alimentary tract as primary points of entry and sites of disease [7,8].

In contrast, pinnipeds are more predisposed to mucocutaneous disease. This pattern may be attributed to specific skin barrier properties, their amphibious lifestyle, involving both wet and dry periods, or distinct patterns of environmental exposure [9,33]. Fungal dermatitis in captive pinnipeds has also been associated with *Yarrowia lipolytica* (previously *Candida lipolytica*), in addition to *Trichophyton mentagrophytes* and *Malassezia* spp [33]. Environmental factors such as excessive chlorination of pool water and elevated water temperatures appear to favor the occurrence of fungal dermatitis in pinnipeds [33,45]. Despite these differences, both taxonomic groups may be affected by gastrointestinal candidiasis, although well-documented case reports are currently limited to cetaceans [7,8,10]. The identification of these species-specific manifestation patterns is essential for achieving early recognition, implementing appropriate diagnostic testing, and initiating specific therapeutic interventions [10]. A detailed comparison of clinical manifestations by anatomical system is provided in Table 2.

## 4. Pathogenesis and Risk Factors

Clinical candidiasis is an emerging disease in marine mammals, necessitating complex interactions among fungal virulence factors, host immune defenses, and environmental stressors [52]. The awareness of these factors is critical in order to create preventive measures and enhance treatment outcomes [44].

### 4.1. Fungal Virulence Factors

**Figure 3:**
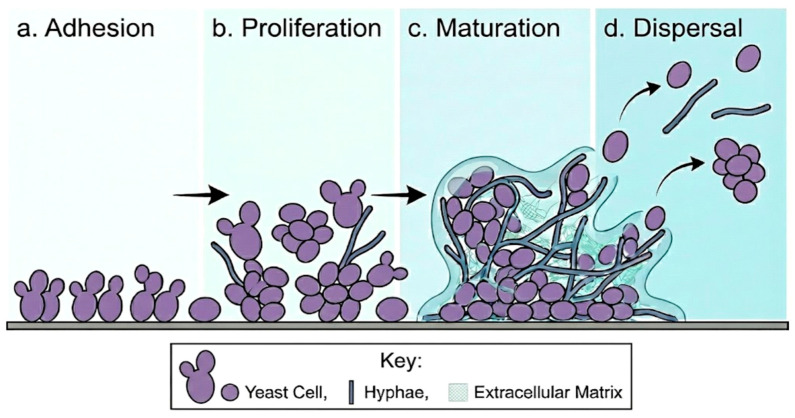
Schematic illustration of successive stages of *Candida* biofilm development: (**a**) the adhesion of yeast cells to biotic or abiotic surfaces; (**b**) proliferation, including cell division and initial formation of hyphae; (**c**) maturation, including the synthesis of the extracellular matrix of both yeast and hyphal morphologies; (**d**) cell dispersal that enables the colonization of new sites.

The *Candida* species have numerous virulence factors that enable colonization, invasion, and pathogenesis [53]. Dimorphic alternation between yeast and hypha is a major virulence factor, as the hyphal form is correlated with the invasion of the tissue in marine mammals and evasion of the immune system [54]. The attachment to host cells and extracellular matrix constituents is performed by adhesins such as the Als (Agglutinin-like sequence) family and Hwp1 (hyphal wall protein 1) [55]. Tissue invasion and acquisition of nutrients occur with the help of the secretion of hydrolytic enzymes such as secreted aspartyl proteinases (SAPs) and phospholipases [56]. Biofilm formation is an important virulence factor, especially in captivity conditions, where marine mammals are likely to be exposed to artificial surfaces, feeding tubes, or medical equipment [57]. Biofilms offer immunity against host immune responses and antifungal therapy and act as persistent reservoirs for the infection [57]. *Candida* cells that are present in biofilms are characterized by increased resistance to antifungal agents and host immune defense [57]. The Candida biofilm is formed in a series of successive stages including adhesion, proliferation, maturation, and dispersal [57] (Figure 3). The yeast originally bound to biotic or abiotic surfaces during adhesion stage via adhesin-mediated interactions. Proliferation is then followed by cell division and early development of hypha resulting in the formation of microcolonies. The maturation stage is marked by the formation of a complex three dimensional structure accompanied by the production of extracellular matrix from both yeast and hyphal morphology, which provide augmented resistance to antifungal agents and host immune defenses. Lastly, dispersal consists of the release of daughter cells, which are able to colonize new sites [58]. It has been recently noted that biofilm-associated *Candida* infections in marine mammals share similar developmental mechanisms [57], in which environmental stressors among captive and wild cetaceans contribute to the transition from commensal colonization to pathogenic infection [10]

**Figure 4:**
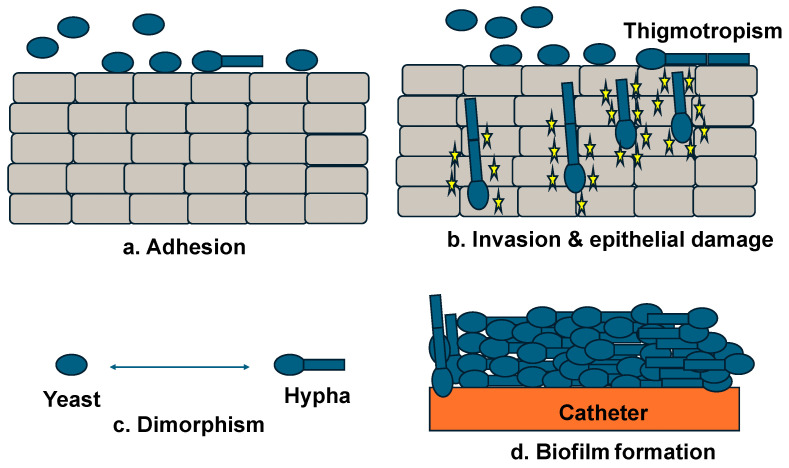
*Candida albicans* pathogenesis: (**a**) adhesion to epithelial surface; (**b**) epithelial contact induces thigmotropism, directing hyphal penetration through breaches with the yellow stars indicating epithelial damage; (**c**) morphological changes: yeast → dimorphism → hypha; (**d**) biofilm formation on catheter showing dense protective structure. Adapted from Haroun et al., Vet. Sci. 2026, 13, 171 under a CC BY license [62].

The contact-dependent morphogenic response of *Candida* to physical contact with epithelial surfaces is known as thigmotropism and is a key virulence mechanism that coordinates the ordered progression of initial adhesion, followed by invasive hyphal growth and biofilm formation [59,60,61] (Figure 4). The absence of nasal turbinates in the respiratory tract of marine mammals, particularly cetaceans, makes the respiratory system especially vulnerable to fungal colonization [19], and this contact-sensing pathway could therefore promote biofilm formation on mucosal surfaces by hyphal breaching of respiratory and gastrointestinal tract tissue.

Environmental research has established that *Candida* species isolated from aquatic habitats share these virulence characteristics. Ramos et al. showed that *Candida* species found in recreational coastal waters exhibited biofilm formation, phospholipase, and proteinase production, as well as antifungal resistance, which indicated the pathogenic capacity of environmental isolates [39]. The isolates attached to abiotic surfaces and formed biofilms to different extents, suggesting that the environmental *Candida* strains have the complete repertoire of virulence factors required to pathogenize a marine mammal host [39]. Biancani et al. found that 100% of *Candida* isolates from both fecal (*n* = 14) and blowhole (*n* = 9) samples of bottlenose dolphins were resistant to all tested azoles, although none of the animals had been treated with antifungal therapy recently [19]. This observation greatly favors the idea that resistance mechanisms can be acquired in the environment, implying that the presence of resistant strains can be propagated in water and can infect marine mammals without direct antifungal selective pressure. Conversely, no resistance was encountered against echinocandin (caspofungin, anidulafungin, and micafungin), meaning that these agents are not contraindicated as treatment [19]. Ferrara et al. recorded a *C. auris* isolate in a dolphin, which was found to be resistant to fluconazole (32 μg/mL by Vitek/Sensititre, >256 μg/mL by Etest) but was susceptible to echinocandins and other new antifungals such as rezafungin, ibrexafungerp, and manogepix. The isolate was placed in clade I and showed a close relationship with the Indian strains based on whole genome sequencing. However, no known *ERG11* (lanosterol 14α-demethylase gene) mutations were found individually. This finding suggests a novel mechanism of azole resistance and warrants further research [18].

It should be noted that the virulence mechanisms described above (Als proteins, SAPs, phospholipases, thigmotropism, biofilm formation) have been primarily characterized through studies on human clinical *Candida* isolates. Their specific roles in marine mammal pathogenesis have not been experimentally confirmed, and their contribution to disease in cetaceans and pinnipeds remains hypothetical. Among the few marine mammal-specific data available, Ramos et al. [39] demonstrated biofilm formation, proteinase, and phospholipase production in environmental *Candida* isolates from coastal waters, and Biancani et al. [19] reported biofilm-related findings in dolphin isolates. However, no in vivo virulence studies have been conducted using marine mammal models, and the immune responses of cetaceans and pinnipeds to *Candida* virulence factors remain largely unexplored.

#### Knowledge Gaps in Marine Mammal-Specific Pathogenesis

Significant knowledge gaps persist regarding *Candida* pathogenesis specific to marine mammals. First, no experimental animal models exist for cetacean or pinniped candidiasis, preventing controlled studies of infection dynamics, immune responses, and therapeutic efficacy. Second, comparative genomic analyses between human and marine mammal *Candida* isolates are lacking, and it remains unknown whether marine mammal strains possess unique virulence gene profiles or adaptations to the marine host environment. Third, the specific immune mechanisms by which cetaceans and pinnipeds respond to *Candida* colonization and invasion are poorly understood [63]. The unique features of cetacean immunity, including the absence of nasal turbinates [7] and the diving-related physiological adaptations, may create distinct host-pathogen interaction dynamics not observed in terrestrial mammals. Fourth, the role of the marine mammal microbiome in modulating susceptibility to *Candida* infections has not been investigated. These gaps highlight the urgent need for dedicated marine mammal-specific pathogenesis research to move beyond extrapolation from human data.

### 4.2. Host-Related Risk Factors

#### 4.2.1. Immunosuppression and Stress

Immunosuppression represents the most significant risk factor for development of invasive candidiasis [64,65]. Stress, whether from captivity, social conflicts, transport, medical procedures, or environmental changes, triggers cortisol release with subsequent immunomodulatory effects [52]. Chronic stress leads to a state of increased susceptibility of opportunistic infections among captive marine mammals [3]. Limited space and altered social structure, unnatural diet and feeding patterns, changes in water quality and chemical treatment, noise and visual disruption by public observation, transport and facility alterations, and loss of social group or offspring are common stress factors in captive environments. Nevertheless, pollution, loss of habitat, food shortage, climate change, ship strikes, and fishing gears entanglement are also significant stressors affecting wild marine mammals and could predispose them to opportunistic infections [3,12]. Cetaceans in captivity are mostly vulnerable to fungal infections, particularly due to stress-related conditions pertaining to confinement and compromised immune response and the issue of increased surveillance in aquatic facilities is crucial [18].

#### 4.2.2. Antibiotic Therapy

Long-term or random administration of systemic antibiotics causes disruption of normal flora in bacteria, which results in the overgrowth of fungi [52]. Several instances of candidiasis infection in marine mammals have been reported after long term antibiotic therapy due to bacterial infections [8]. This association is similar to effectively documented trends in human and veterinary medicine [44].

#### 4.2.3. Concurrent Diseases and Co-Infections

Moreover, comorbidities may also be a contributing factor to the risk of fungal infections such as candidiasis [66]. Pre-disposing factors include bacterial pneumonia, skin trauma, and parasitic infections, in addition to neoplasia. Immunosuppression via viral diseases, especially morbillivirus, can predispose one to secondary fungal infections [67]. The example of respiratory co-infection in the bottlenose dolphin with *Nakaseomyces glabratus* (formerly *Candida glabrata*) and parainfluenza virus demonstrates the nature of polymicrobial infections in marine mammals [66].

### 4.3. Environmental Factors

#### 4.3.1. Water Quality and Chemical Treatments

Water disinfection practices in captive facilities has a significant effect on the fungi ecology [3]. Disproportionate levels of chlorination may destroy the beneficial microbes and cause fungal overgrowth. Over-chlorination of swimming pool water has also been detected as another important environmental process associated with the development of fungal dermatitis in captive pinnipeds [33]. Elevated water temperatures may promote *Candida* growth [38]. Appropriate water chemistry in terms of pH, salinity, and organic load is vital in disease prevention [33].

#### 4.3.2. Environmental Contamination

Human-related *Candida* species in wild marine mammals are a sign of environmental contamination due to anthropogenic factors [52,68]. Sewage discharge, coastal pollution, and agricultural runoff can introduce *Candida* species and antifungal-resistant strains into marine ecosystems [38,39]. Further, changes in temperature and chemistry of the ocean due to climate changes can potentially affect fungal distribution and virulence [68].

#### 4.3.3. Heavy Metal Exposure

Opportunistic fungal infections like candidiasis can be a threat to marine mammals due to environmental contaminants [69,70]. Mercury and polychlorinated biphenyls (PCBs) are potent immunosuppressants in marine mammals, and have an established impact on lymphocyte proliferation, phagocytosis and innate and adaptive immunity [13]. Elevated levels of these contaminants in tissues have caused immune dysregulation, which may predispose the immune system to opportunistic infections [71]. Marine pollution caused by chemicals could impair the immune system of cetaceans, making them susceptible to colonization by opportunistic fungal pathogens like *Candida* species [3]. More recent findings indicate that environmental stress factors, coupled with pollution and pharmaceutical contamination in seawater, have the potential to cause additional development of antifungal-resistant *Candida* species in cetaceans, representing another marine mammal health issue [68].

## 5. Antifungal Resistance—An Emerging Threat

Antifungal resistance among *Candida* species of marine mammals is an emerging concern that warrants further investigation across broader populations [68,72]. This effect is similar to the global trends in human and veterinary medicine, where the widespread use of antifungal drugs has contributed to the selection of resistant strains [73,74]. The Fungal Priority Pathogens List was first compiled by the World Health Organization in 2022, which named *Candida albicans* and *C. auris* as critical priority pathogens [75].

*Candida* resistance patterns are also threatening as evidenced by environmental sources. Ramos et al. in their study of recreational coastal waters of Rio de Janeiro showed that the *C. tropicalis* isolates were resistant to azoles and susceptible to amphotericin B, flucytosine, and caspofungin. *P. kudriavzevii* (*C. krusei*) isolates were resistant to fluconazole, caspofungin, and itraconazole, with 42.8% also resistant to flucytosine [39]. Such distributions of environmental resistance are also comparable with that observed in cetacean isolates and suggest that marine mammals can acquire a resistant strain in contaminated water resources [39]. Garcia-Bustos et al. found that over 86 and 80 percent of *C. albicans* and *C. tropicalis* isolates of captive dolphins, respectively, were resistant or dose-dependently susceptible (R or SDD) to fluconazole, and itraconazole resistance or SDD rates were 80 and 80 percent in each species, respectively [10] (Table 3).

### 5.1. Azole Resistance

Azole antifungals, such as fluconazole, itraconazole, and voriconazole represent the first line of treatment for candidiasis because of their broad spectrum and good pharmacokinetics, as well as their oral formulations [77]. Nevertheless, cases of resistance to azoles have been reported more frequently in the isolates of marine mammals [14,15]. In 2019, at the Port of Nagoya Public Aquarium, four of thirteen *Candida* isolates from fourteen bottlenose dolphins were resistant to azole antifungals. Interestingly, certain isolates were resistant with no previous exposure to these agents in individual animals, thereby indicating environmental originality of resistant strains or horizontal transmission of resistance mechanisms [14]. Shirakata et al. reported that all eight *C. albicans* isolates from dolphin blowholes in Enoshima, Japan, were resistant to fluconazole, itraconazole, and voriconazole, while remaining susceptible to amphotericin B and micafungin [15]. Previously, Ohno et al. discovered that *C. tropicalis* isolates in the Port of Nagoya Public Aquarium were resistant to both itraconazole and voriconazole. Three of four N. glabrata isolates were resistant to itraconazole, consistent with the intrinsic reduced azole susceptibility of this species [14]. Another study, conducted by Biancani et al. on bottlenose dolphins in Italy in 2025, showed concerning findings since 100% of *Candida* isolates in fecal (*n* = 14) and blowhole (*n* = 9) samples were totally resistant to all the tested azoles [19]. The identified species were *C. albicans*, *C. tropicalis*, *N. glabratus* (formerly *Candida glabrata*), and *C. parapsilosis*. More importantly, none of the tested animals had undergone antimycotic treatments in the recent past, which is a great indication of resistance being acquired in the environment. The absence of antifungal treatment was documented by Biancani et al. [19] based on facility medical records, although this documentation may not account for treatments prior to the animals’ arrival at the facility. However, the absence of direct antifungal treatment does not exclude indirect environmental exposure to azole compounds through agricultural runoff, wastewater contamination, or other anthropogenic sources, which may exert selective pressure on commensal *Candida* populations. Conversely, resistance to echinocandins was not observed (caspofungin, anidula-fungin, and micafungin). However, a study in Japan highlighted that 60.4% of *C. tropicalis* isolates from various sources showed intermediate susceptibility to caspofungin [76], indicating that susceptibility to these agents should continue to be closely monitored [19]. Of special interest, the first case of *C. auris* colonization was reported in a captive dolphin in the Dominican Republic by Ferrara et al., which coincided with a *C. auris* bloodstream infection found in a human being in the same geographic location [18]. The dolphin isolate was fluconazole resistant (MIC, minimum inhibitory concentration, 32 μg/mL by the automated system Vitek 2; >256 μg/mL by Etest gradient diffusion method) but was still susceptible to echinocandins and new antifungals such as rezafungin, ibrexafungerp, and manogepix. Whole genome sequencing assigned the isolate to clade I, which is closely related to Indian strains, but, unexpectedly, did not identify any of the known mutations in ERG11, which implies a new mechanism of azole resistance [18]. The mechanisms of azole resistance in isolates of marine mammals include candida drug resistance (CDR) efflux pump overexpression (CDR2, CDR3), multidrug resistance 1 (MDR1) overexpression, target enzyme (ERG11 mutations), and ergosterol biosynthesis pathways change [74,78]. Other strains are multidrug resistant, making therapeutic decisions more difficult [74].

The resistance data reported above should be interpreted with caution, considering the limited sample sizes and single-facility origin of each study. Specifically, Ohno et al. [14] reported azole resistance in 4 of 13 *Candida* isolates from 14 dolphins at a single Japanese aquarium (Port of Nagoya Public Aquarium), using the CLSI (Clinical and Laboratory Standards Institute) M27-A3 broth microdilution method with breakpoints for itraconazole (S ≤ 0.125, SDD 0.25–0.5, R ≥ 1 µg/mL) and voriconazole (S ≤ 1, R ≥ 4 µg/mL) [14]. Shirakata et al. [15] reported azole resistance in all 8 *C. albicans* isolates from dolphin blowholes at a single facility (Enoshima Aquarium, Japan), using the CLSI M27-A3 broth microdilution method with the same breakpoints for itraconazole (R ≥ 1 µg/mL) and voriconazole (R ≥ 4 µg/mL); all isolates were susceptible to amphotericin B and micafungin [15]. Biancani et al. [19] reported 100% azole resistance in all *Candida* isolates from the fecal (*n* = 14) and blowhole (*n* = 9) samples of seven bottlenose dolphins at a single Italian facility (Oltremare, Riccione), using the CLSI M27 broth microdilution method with CLSI M60 clinical breakpoints for classification of susceptible, intermediate, and resistant categories [19]. Takahashi et al. [25], as reviewed by Garcia-Bustos et al. [10], reported high rates of fluconazole resistance or dose-dependent susceptibility (R/SDD) in *C. albicans* (86.7%, 13/15 isolates) and *C. tropicalis* (80%, 8/10 isolates) from captive dolphins at the Okinawa Churaumi Aquarium, using CLSI M27-A3 broth microdilution with breakpoints for fluconazole (S ≤ 8, SDD 16–32, R ≥ 64 µg/mL) and itraconazole (S ≤ 0.125, SDD 0.25–0.5, R ≥ 1 µg/mL) [10,25]. None of these studies established clinical correlation between in vitro resistance and therapeutic failure. Given that all data originate from individual aquaria in Japan and Italy, these findings represent facility-specific resistance profiles and should not be extrapolated to global trends without larger-scale, multi-center surveillance studies employing standardized methodologies.

#### Molecular Mechanisms of Azole Resistance

The molecular mechanisms underlying azole resistance in *Candida* species from marine mammals involve several well-characterized pathways. ERG11 mutations, affecting the lanosterol 14α-demethylase enzyme (the target of azole antifungals), can reduce drug binding affinity and confer resistance [74,78]. Overexpression of efflux pumps, particularly ATP-binding cassette (ABC) transporters such as CDR1 and CDR2 (*candida* drug resistance) and the major facilitator superfamily (MFS) transporter MDR1, actively expels azole drugs from the fungal cell, reducing intracellular drug concentrations [74,78]. Transcription factor alterations, including gain-of-function mutations in TAC1 and UPC2, upregulate efflux pump expression and ergosterol biosynthesis genes, respectively [74]. Khalifa et al. demonstrated that azole-resistant *C. tropicalis* isolates from Japan, including those from marine aquarium environments, exhibited elevated expression of CDR2, CDR3, TAC1, UPC2, and the ergosterol biosynthesis gene HMG, confirming these mechanisms in marine mammal-associated strains [76]. No FKS1 hot spot mutations (conferring echinocandin resistance) were detected [76]. Biofilm-related tolerance represents an additional mechanism whereby *Candida* cells embedded in mature biofilms exhibit 100–1000-fold increased resistance to antifungals compared with planktonic cells, due to restricted drug penetration and metabolic heterogeneity within the biofilm structure [57].

It is critical to distinguish between intrinsic and acquired resistance when interpreting antifungal susceptibility data from marine mammals. Certain species possess inherent, species-level resistance: *Pichia kudriavzevii* (formerly *Candida krusei*) is intrinsically resistant to fluconazole due to structural alterations in its ERG11 gene [74], while *Nakaseomyces glabratus* (formerly *Candida glabrata*) exhibits intrinsic reduced susceptibility to azoles and can readily acquire further resistance [74]. In contrast, acquired resistance develops through de novo mutations or gene expression changes in response to selective pressure, whether from clinical antifungal use or environmental exposure to agricultural azole fungicides [73]. The observation of azole resistance in marine mammal isolates without documented antifungal treatment history raises the hypothesis of environmental acquisition; however, indirect selection through agricultural runoff, wastewater contamination, or other environmental sources of azole compounds cannot be excluded and warrants further investigation.

Notably, the *C. auris* isolate reported by Ferrara et al. [18] from a captive dolphin in the Dominican Republic was fluconazole-resistant but lacked known ERG11 mutations, suggesting a novel mechanism of azole resistance that warrants further molecular characterization. Regarding MIC methodology and interpretation, it should be emphasized that the clinical breakpoints established by CLSI [79] and EUCAST (European Committee on Antimicrobial Susceptibility Testing) [80] were developed using human pharmacokinetic and pharmacodynamic data and may not directly translate to marine mammal species. Species-specific antifungal breakpoints for cetaceans and pinnipeds do not currently exist, and MIC values must therefore be interpreted with caution, ideally in conjunction with species-specific pharmacokinetic data when available, such as the voriconazole pharmacokinetic study in bottlenose dolphins by Ferrier et al. [81].

### 5.2. Clinical Implications

Antifungal resistance has great clinical consequences on the health of marine mammals [68]. Not all cases of treatment have been successful [7]. Agents resistant to infections have very limited therapeutic options and instead require the use of more toxic agents (amphotericin B) or newer antifungals (echinocandins) with unknown safety in marine mammals [44,77].

The development of resistance also casts significant concerns regarding the antifungal stewardship in the veterinary domain and the possibility of selecting the resistant strains in aquatic environments [17]. The prophylactic use of the antifungal agent in the context of stressful situations (transport, medical operations) could lead to resistance development and must be estimated against possible advantages.

### 5.3. Environmental Reservoirs of Resistance

The aquatic environment may behave as a reservoir for antifungal-resistant *Candida* species [38,39]. Environmental resistance can be caused by agricultural application of azole fungicides, pharmaceutical contamination of waterways and the survival of resistant waterways in biofilms [68]. Exposure of wild marine mammals to such environmental reservoirs may result in acquisition of resistant strains in the absence of clinical antifungal exposure, and this may explain the resistance in animals with no treatment history [82].

The direct evidence of environmental reservoirs was also made as *Candida* species were identified in dolphin tank pools water samples by the culture and fluorescent in situ hybridization (FISH) methods [19]. In the majority of the sampling points, *C. albicans* was found, whereas *C. tropicalis* and *C. parapsilosis* were identified in certain locations. The antifungal susceptibility of the *C. tropicalis* isolate in the environment was also similar to that of the isolate found in dolphins, which confirms the hypothesis of environmental spread of resistant *Candida* species [19].

## 6. Diagnosis and Treatment

The accurate diagnosis and proper treatment of candidiasis in marine mammals remain a challenging issue due to non-specific clinical manifestations, problems with gathering of samples, and deficiency in pharmacokinetic data of antifungal drugs in those species [19].

### 6.1. Diagnostic Approaches

#### 6.1.1. Clinical Examination and Imaging

Primary examination is comprised of extensive physical examination, and mucosal surface, respiratory, and behavioral alterations [8]. The endoscopy can be used to visualize the lesions of the blowhole, esophagus, and stomach directly and to obtain samples to be further cultured and examined with histopathology [8]. Pulmonary infiltrates, organomegaly, or other evidence of systemic infection may be detected using diagnostic imaging (radiography, ultrasonography, etc.).

#### 6.1.2. Cytology and Histopathology

Swabs or brushings or tissue impressions examined cytologically can give rapid presumptive diagnosis by identification of yeast cells and pseudohyphae [7]. Periodic Acid-Schiff (PAS) and Grocott’s Methenamine Silver (GMS) are special stains that are useful to increase visualization of fungal components in tissue sections. Histopathology demonstrates the typical aspects of candidiasis such as granulomatous inflammation, tissue necrosis, yeast and hyphal forms invasion [7,8].

#### 6.1.3. Culture and Identification

Cultures of fungi are the gold standard of a definitive diagnosis and can be used to identify the species and perform an antifungal susceptibility test [14,25]. Samples of various locations (blowhole, oral, feces, lesions) are to be taken on a selective media (Sabouraud dextrose agar including antibiotics). Molecular techniques have mostly replaced traditional techniques such as colony morphology and biochemical reactions in identification. Matrix-Assisted Laser Desorption/Ionization Time-Of-Flight Mass Spectrometry (MALDI-TOF MS) has become a rapid and reliable tool to identify yeasts in marine mammals, although misidentification of new species like *C. auris* continues to be a challenge that needs confirmatory molecular analysis [18]. Marques et al. designed a practical in-house respiratory culture protocol of bottlenose dolphins at Zoomarine Portugal, implementing quarterly screening of sputum samples as part of the preventive medicine program [44]. The protocol involved the use of Sabouraud Dextrose Chloramphenicol Agar (SDCA) and CHROMagar *Candida* Plus, enabling differentiation of *C. albicans*, *N. glabratus (C. glabrata)*, *C. tropicalis*, *P. kudriavzevii (C. krusei)*, and *C. auris* [41].

#### 6.1.4. Molecular Diagnostics

**Figure 5:**
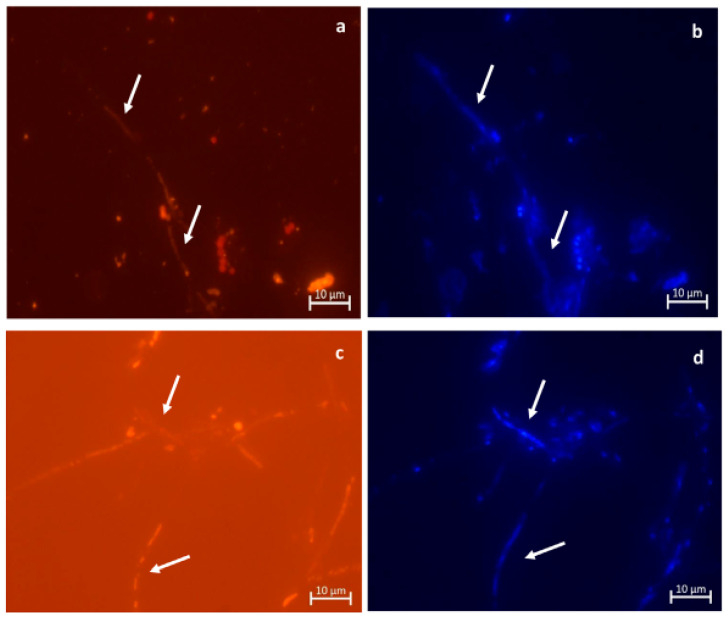
FISH of *Candida tropicalis* labelled with specific probe (**a**,**c**) and DAPI (**b**,**d**). Panels (**a**,**b**) show sample ‘C’; panels (**c**,**d**) show the control. Reproduced with permission from Biancani et al. (2025), *Veterinary Medicine and Science*, 11(6): e70668 [19].

The identification of the species through molecular methods is fast and highly accurate [25]. The internal transcribed spacer (ITS) of ribosomal DNA sequencing is the definitive method for species identification and has been successfully applied to cases of marine mammals [15,25]. However, the accuracy of the atypical or environmental *Candida* species can be jeopardized by some of the database limitations. Sound identification of species is important because traditional automated systems might not identify the emerging pathogens correctly. According to Ferrara et al., both human and dolphin *C. auris* isolates were wrongly identified as *Meyerozyma guilliermondii* by the Vitek XL system, and MALDI-TOF MS identified *C. auris* with high confidence scores [18]. This highlights the role of advanced diagnostic tools especially in the multidrug-resistant emerging pathogens in the marine mammals [18]. Another molecular technique is fluorescence in situ hybridization (FISH), which enables quick and selective observation of *Candida* species in both clinical and environmental specimens (Figure 5). The method is specifically useful in identifying pathogens in water samples of holding facilities, as demonstrated by Biancani et al., who managed to detect *C. albicans*, *C. parapsilosis* and *C. tropical* is in aquatic environments of bottlenose dolphin facilities using species-specific probes [19].

#### 6.1.5. Antifungal Susceptibility Testing

All clinically significant isolates should undergo susceptibility testing in the context of the development of the antifungal resistance [14,15]. Standardized broth microdilution techniques can give minimum inhibitory concentration (MIC) values, which are used to make therapeutic decisions [73]. Nevertheless, the clinical breakpoints that have been set using human isolates might be inapplicable to the medical treatment of marine mammals, and the results are to be interpreted with reference to the species-specific pharmacokinetic and treatment effect. According to Marques et al., the antifungal susceptibility test (AFST) has become relevant in fungal infection treatment in cetaceans [41]. Their internal protocol involved AFST that was done prior to the onset of treatment and during treatment to ensure there is no resistance development. The authors pointed out that extended courses of antifungals can cause a change in resistance patterns, and continuous monitoring of the susceptibility is necessary [41]. Antifungal susceptibility testing can be accomplished via several standardized methods available with marine mammals’ isolates. Yeast testing is still conducted with reference to the Clinical and Laboratory Standards Institute (CLSI) method of broth microdilution (M27, 4th edition) [79]. Alternative standardized protocols that have species-specific clinical breakpoints are proposed by the European Committee on Antimicrobial Susceptibility Testing (EUCAST) [80]. These standardized methods have been applied to cetacean isolates [18,19].

Marques et al. performed antifungal susceptibility testing using Etest strips for itraconazole, fluconazole, and voriconazole, demonstrating the importance of regular mycological monitoring in managed cetaceans [41].

**Figure 6:**
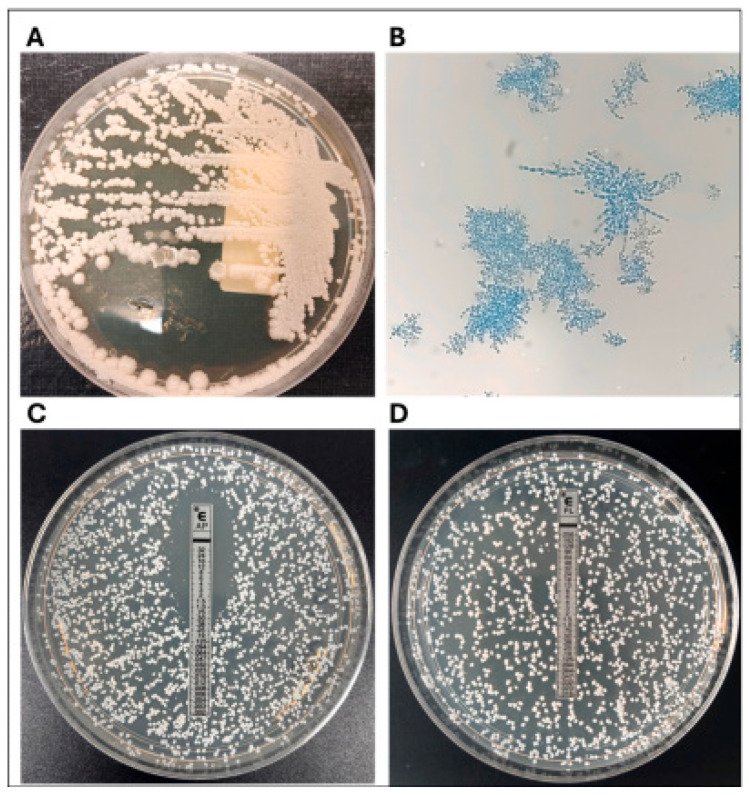
Morphological and antifungal susceptibility features of *Candida* (*Candidozyma*) *auris* isolate from captive dolphin (Case 2). (**A**) Macro-morphology: colonies appear dull and dry with margins ranging from smooth to lobed, exhibiting wrinkled surface texture on Sabouraud dextrose agar. (**B**) Micromorphology: yeast cells appear with long-grain rice morphology, which are organized into chains of cells typical of *C. auris*. (**C**) Amphotericin B (AP) Etest reading pattern, concentration on the strip in micrograms per milliliter. (**D**) Fluconazole (FL) Etest reading pattern, with concentration on the strip in micrograms per milliliter. Figure reproduced from Ferrara et al., *Medical Mycology Case Reports*, 50, 100745, under a CC BY license [18].

Marine mammals isolates have been tested using commercial systems. Ferrara et al. performed *Candida auris* susceptibility testing on Vitek^®^ XL (AST-YST 08 card), Etest, and Sensititre Yeast One. The human isolate (Case 1) showed: fluconazole > 64 μg/mL, voriconazole 1 μg/mL, caspofungin 0.25 μg/mL, micafungin 0.12 μg/mL, and amphotericin B 0.5 μg/mL. The dolphin isolate (Case 2) showed: fluconazole 32 μg/mL, voriconazole 0.12 μg/mL, caspofungin 0.12 μg/mL, micafungin 0.06 μg/mL, and amphotericin B 0.5 μg/mL [18]. Further tests were done on the dolphin isolate with newer antifungals such as rezafungin (0.03 μg/mL), ibrexafungerp (0.06 μg/mL) and manogepix (<0.06 μg/mL) by CLSI BMD M27 [18]. The antifungal and morphological susceptibility profiles of *C. auris* isolates from marine mammals are representative of the multidrug resistance patterns, which has been increasingly cataloged in dolphins (Figure 6). These results highlight the significance of proper species identification and extensive susceptibility testing of candidiasis in captive cetaceans in clinical practice [18].

Biancani et al. used broth microdilution checkerboard technique to assess the synergistic activity between antifungal combinations (fluconazole/terbinafine, voriconazole/terbinafine, voriconazole/nystatin), computing the indices of the fractional inhibitory concentration (FIC) between drugs. Their findings revealed that 100% of fecal isolates were resistant to azoles, 50 percent were resistant to 5-flucytosine, and 21.4 percent were resistant to amphotericin B, with none having an echinocandin resistance [19]. Susceptibility results should be interpreted with caution because clinical breakpoints developed on human isolates do not necessarily apply to marine mammals because of differences in pharmacokinetics, drug distribution, and attainable tissue concentrations. Marine mammal *Candida* isolates have not been established to have species-specific breakpoints necessitating the comparison of MIC values, with clinical outcomes and pharmacokinetic values when available [41].

### 6.2. Treatment Strategies

#### 6.2.1. Antifungal Therapy

The treatment procedures need to be personalized, depending on the severity of the disease, the identification of the species, the susceptibility findings, and the size of the animals. Ketoconazole (2.5 mg/kg per os (PO) twice daily (BID) for 18 days in *T. truncatus*, followed by levamisole 9 mg/kg biweekly; up to 12 mg/kg once daily (SID) or BID for 14–21 days in *D. leucas* [8]; 5.5–11 mg/kg daily for 77 days [24] was once successfully used in early cases but today has been mainly superseded by newer azoles because of hematological toxicity, including severe pancytopenia [24]. Supplementation with prednisolone (0.01 mg/kg PO SID) may be appropriate to compensate for ketoconazole inhibition of glucocorticoid production [51]. Candidiasis generally responds well to ketoconazole, itraconazole, and echinocandins [51]. Presently, fluconazole (2.5–5 mg/kg BID in *T. truncatus* [7] is widely used, but resistance patterns are on the rise, which limits its application [14,15,19]. Alternatively, itraconazole (2.0–2.5 mg/kg BID PO [14]; 2.5–5 mg/kg BID in *T. truncatus* [7] has a wider spectrum than fluconazole and is active against yeasts and molds [7]. A major obstacle in the treatment of non-*albicans Candida* species is their innate resistance to older-generation azoles and even echinocandins, in many cases leaving voriconazole or posaconazole as the main choices for therapy [7]. Ferrier et al. [81] established a voriconazole dosing regimen in bottlenose dolphins consisting of a 10 mg/kg loading dose divided in three administrations (3.3 mg/kg every 24 h), followed by a 4 mg/kg weekly maintenance dose, with a therapeutic window of 1–5 mg/L. Due to a half-life of approximately 11 days in dolphins (compared to 6 h in humans), therapeutic drug monitoring (TDM) is mandatory. Without TDM, 15–17% of dolphins did not reach the therapeutic window of 1–5 mg/L without TDM, and 38% did not reach the narrower 2–4 mg/L target range [81]. Voriconazole hepatotoxicity was observed at plasma concentrations > 5 mg/L, with elevated transaminases in 69% (11/16) of samples above this threshold [81]. Visual adverse events (eye squeezing) were reported in one dolphin at a simulated plasma concentration of 3.8 mg/L [81]. Ohno et al. [14] also used voriconazole with an intermittent medication regimen (loading dose 1.0–2.4 mg/kg BID PO for 3 consecutive days; maintenance dose 1.0–3.1 mg/kg BID PO every 7–10 days) and monitored plasma concentrations to avoid toxic levels. In one case of oral and esophageal candidiasis in bottlenose dolphins, voriconazole at 3 mg/kg PO BID for 5 days was extremely effective but provoked a rise in lactate dehydrogenase (LDH). Silymarin (Legalon) was added at 300 mg BID as hepatoprotective support [7]. Simeone et al. [23] reported a case of bilateral *C. albicans* keratomycosis in a bottlenose dolphin treated with oral fluconazole (800 mg PO q 24 h, subsequently increased to BID) combined with topical and subconjunctival voriconazole 1%, which resulted in clinical resolution. Amphotericin B can be a viable option in case of severe or resistant infections; however, because of nephrotoxicity issues, amphotericin B should only be used sparingly [8]. Intravenous administration of liposomal amphotericin B reportedly induced renal dysfunction in a bottlenose dolphin [14]. Oral amphotericin B (2 mg/kg three times daily (TID) for 48 days) was used to treat *Candida* gastritis in a dolphin [15]. In marine mammals, the liposomal form nebulized at 25 mg in 5 mL of sterile water BID has given promising results in some refractory respiratory infections, minimizing the side effects of systemic therapy [7]. Echinocandins (caspofungin, micafungin, anidulafungin) are effective against *Candida* spp. and may maintain activity against azole-resistant isolates [7]; however, intravenous administration of micafungin in a dolphin reportedly caused leukopenia [14]. No resistance to echinocandins was observed in *Candida* spp. isolated from captive dolphins [19]. Flucytosine (20 mg/kg TID in *T. truncatus* [7] is active against *Candida* spp. but must be combined with another antifungal agent because of the rapid emergence of resistance when used alone [7]. Terbinafine (2–4.5 mg/kg SID/BID in *T. truncatus* [7] has expanded use due to its broad antifungal spectrum [7]. Nystatin (7000–14,000 IU BID/TID [7] is considered a relatively safe drug for treating oral or gastrointestinal candidiasis [7], although it is not absorbed across intact skin or mucous membranes [7]. In cases of long-term antibiotic therapy in marine mammals, nystatin is frequently given to prevent overgrowth of gastrointestinal yeasts [7]. Nystatin and miconazole are absorbed poorly or not at all when administered orally and are of limited effectiveness against skin lesions [8]. Recent studies have shown that combination therapy could help to treat the infections in dolphins, which are resistant to azoles. The combination of triazoles and terbinafine shows promise for treatment of resistant *Candida* infections [7]. Biancani et al. assessed the synergistic effects of fluconazole with terbinafine through the checkerboard method and have observed synergistic effects in 71.4% of fecal isolates and 55.6% of blowhole isolates, and additive effects in the rest [19]. Voriconazole and terbinafine were found to have additive effects only (71.4–77.8%), whereas the effect of voriconazole and nystatin was antagonistic in the majority of isolates (66.7–71.4) and should not be used. Triazole and polyene combinations may be antagonistic [7]. Notably, a dolphin which presented with clinical features of gastrointestinal candidiasis was treated successfully using the fluconazole-terbinafine combination, and full resolution was achieved, as evidenced by negative culture follow-ups [19].

#### 6.2.2. Adjunctive Therapies

Levamisole hydrochloride (9 mg/kg) has been administered with antifungals as an immune potentiating agent [8,11] Levamisole seems to be effective in enhancing immunocompetence in already immunosuppressed dolphins but will not change the immunocompetence of a normal animal [11]. A full recovery requires intensive supportive treatment like fluid therapy, nutritional support and treatment of comorbid bacterial infections as was demonstrated in the treatment of a beluga whale with concurrent candidiasis and bacterial pneumonia that necessitated antibiotics, cimetidine, antacids, and tube-feeding [8]. Supportive therapy with lithium carbonate and oxymethalone was also used to manage ketoconazole-induced pancytopenia in a dusky dolphin [24]. New methods, such as allogeneic adipose-derived stem cells and autologous platelet-rich plasma, have been used in instances of ocular candidiasis, with clinical resolution of bilateral *C. albicans* keratomycosis in a bottlenose dolphin [23].

#### 6.2.3. Treatment Duration and Monitoring

Dunn et al. reported that the treatment of candidiasis with ketoconazole over 18 days followed by 8 biweekly oral doses of levamisole at 9 mg/kg in a bottlenose dolphin resulted in regression [8]. In another case, Fothergill et al. reported that ketoconazole treatment was continued for 77 days at 5.5–11 mg/kg daily in a dusky dolphin with clinical improvement [24]. On the other hand, Biancani et al. reported that the combination of fluconazole-terbinafine therapy led to a complete resolution confirmed by two biweekly negative fecal cultures [19]. Marques et al. recommended that serial fungal cultures and antifungal susceptibility testing (AFST) should be performed before starting any antifungal treatment and throughout therapy, as there may be a shift in the resistance pattern of the causative agent during prolonged antifungal therapy; they further suggested follow-up sampling every 15 or 30 days during treatment [41]. Fothergill et al. [24] further emphasized that hematologic surveillance is a requisite, as ketoconazole-induced pancytopenia was documented in their cases, and that premature discontinuation of antifungal therapy often leads to relapse, as demonstrated in a female dusky dolphin, where withdrawal of ketoconazole treatment caused recurrence of severe candidiasis, requiring retreatment at a lower dosage of 2.6 mg/kg/day; however, even at a subsequently increased dosage of 5.2 mg/kg/day, neutropenia and thrombocytopenia developed again within 7 days, requiring permanent discontinuation [24]. Furthermore, Pollock et al. documented recrudescence of fungal dermatitis, including candidiasis, even after treatment in captive pinnipeds, where recrudescence of disease was common and resolution of signs also occurred spontaneously without treatment [33].

### 6.3. Challenges and Limitations

There are several challenges and limitations in the management of candidiasis in marine mammals. The limited antifungal pharmacokinetic information on marine mammals has not been published; in practice, cetacean doses are commonly calculated by scaling of human doses that, in the absence of therapeutic drug monitoring, may lead to underdose or toxicity due to significant interspecies variations in half-life and distribution volume of drugs [81]. No antifungal breakpoints of mold have been determined in cetaceans [19]. The variability in drug absorption, in particular, applies to the case of azole antifungals, where the intake of food (in particular, the fatty fraction of the diet) and gastric pH can have pronounced impact on the bioavailability [81]. Due to these reasons, therapeutic drug monitoring of voriconazole, itraconazole and posaconazole is recommended at least in non-acute and/or complicated cases [7]. Current treatment protocols in cetacean candidiasis are still highly dependent on the early case reports of the 1980s [8,24], and no controlled clinical trials or standardized treatment guidelines have been established for antifungal therapy in marine mammals. The majority of therapeutic choices follow isolated case reports, case series, and clinical experience instead of being evidence-based procedures [7]. Outcome data are insufficient to justify the application of synergy testing to predict the most effective antimicrobial combinations to use in vivo [19]. Antifungal susceptibility test though a valuable instrument in the choice of suitable therapy is not universally practiced in aquaria [19]. Molecular methods to circumvent the species detection and identification shortcomings of traditional biochemical procedures are neither commonly accessible nor uniformly applied across studies [10]. The complexity of determining whether *Candida* is the main cause of an infection or a contaminant or just a colonizer makes the results interpretation more complex [10]. Cetaceans that are held captive are also vulnerable to opportunistic infections especially because of the stress factors that are linked to confinement and immune system breakdown [10]. Moreover, the development of antifungal resistance in cetacean isolates is an increasing problem in the treatment management with cetacean isolates showing a high level of resistance to fluconazole and itraconazole and some isolates having resistance to several classes of antifungal agents [10]. Pollution of seawater by environmental and pharmaceutical agents and the indiscriminate application of antifungals in agriculture may be implicated in the increasing frequency of antifungal resistance [10]. The possibility of the evolution of extremely resistant and virulent species like *C. auris* in marine mammals also promotes the urgency of the problem [10,18].

## 7. One Health Perspective and Zoonotic Potential

The One Health concept recognizes the interconnectedness of human, animal, and environmental health [16]. An example of such relationships is candidiasis in marine mammals with several pathways, including zoonotic transmission, shared environmental exposure, and antimicrobial resistance [17].

Examples of such connections include candidiasis in marine mammals, which Bossart highlighted, with cetaceans as important ecologic indicators of marine ecosystem health, and *Candida* infections in wild animals as indicators of environmental changes, pollution levels, and other disturbances in the whole ecosystem [3]. The extensive use of antifungals in large-scale agribusiness and livestock farming coupled with incorrect disposal of antifungal drugs in wastewater treatment plants has led to the appearance of resistance in aquatic yeasts [68]. The pathogenicity of different *Candida* species has also been demonstrated to respond to more severe environmental conditions in the context of global warming, which could be causing the appearance of high-resistance and virulent *Candida* species [4,5]. A thorough understanding of the ecology of these pathogens in cetaceans in an interdisciplinary and collaborative The One Health approach is vital in formulating preventive strategies that protect the health of marine mammals as well as human health [16].

### 7.1. Zoonotic and Reverse Zoonotic Transmission

The potential bidirectional transmission of *Candida* species between humans and marine mammals has significant implications for public health [25,76]. The occupational risks of exposure to resistant fungal pathogens may be present in personnel that works in marine mammal facilities such as veterinarians, trainers, and aquarists [83,84]. On the other hand, *Candida* strains related to human beings can be introduced to the population of the captive marine mammals by human contacts, contamination of equipment, or by the water system within the facility. Although the direct expression of candidiasis between marine mammals and humans was not reported, the detection of human-associated *Candida* species in marine mammals, including captive ones and wild ones, implies that microorganisms are being exchanged [25,27]. The workers are expected to practice proper biosecurity such as hand hygiene, personal protective gear, and environmental decontamination [85].

Molecular epidemiological studies have provided evidence for cross-species transmission of *Candida* between marine mammals and humans. Takahashi et al. determined the same genotypes of *C. albicans* in a dolphin and a staff member working as a veterinarian, according to molecular typing based on the combination of the MDR1 gene and ITS rDNA sequences [25]. On the same note, Khalifa et al. revealed that *C. tropicalis* isolated in patients and marine mammals in Japan shared the same sequence types (ST232 and ST933), indicating cross-boundary dissemination and cross-transmission of *C. tropicalis* isolates [76]. These results point to the significance of biosecurity and comprehensive surveillance systems including marine mammal and human health in a single program [25,76].

### 7.2. Marine Mammals as Sentinel Species

Marine mammals are also the best sentinel species to monitor the health of an ecosystem due to their long lifespan, high trophic levels, and the accumulation of environmental contaminants [3]. The existence and nature of fungal infections in these animals can be indicative of more general environmental transformations, such as the introduction of antifungal agents and antipathogenic microbes into marine environments, the impact of climate change on marine microbial communities and disease ecology, the influence of pollution on immune systems and susceptibility to illness, and the impact of anthropogenic stressors on the health of wildlife at the population scale. Surveillance of fungal diseases in marine mammals can be used to give early warning of new threats that might eventually impact on human population or to show declining environmental conditions that will need to be addressed [86].

### 7.3. Antifungal Resistance as a One Health Issue

The development of *Candida* resistance to antifungal drugs in marine mammals is also associated with concerning patterns observed in human healthcare [68,75]. The introduction of azole fungicides in farming can cause the selection of cross-resistant human and animal pathogens due to environmental pollution [7]. The pharmaceutical pollution of waters leads to the introduction of clinical antifungals into water ecosystems, which may cause resistance in the environmental population of the fungus [87]. To handle the issue of antifungal resistance, One Health strategies such as stewardship initiatives in human and veterinary healthcare, regulated use of agricultural fungicides, better treatment of wastewater to eradicate pharmaceutical wastes, and surveillance systems to track the resistance trends across species and settings are necessary [17,82].

Biancani et al. emphasized that antifungal susceptibility testing should be conducted routinely in aquaria and should include both symptomatic cases and asymptomatic carriers, to facilitate early detection of resistant strains and guide targeted mitigation measures [19]. Antifungal agents other than azoles can be used as a first-line therapy of candidiasis when the presence of the azole-resistant *Candida* species is confirmed in a facility. The paper also highlighted that azole resistant *Candida* species may be directly or indirectly transmitted to humans, which justifies the necessity of combined surveillance and control strategies [19].

The discovery of *Candida auris* in a captive dolphin by Ferrara et al. in the Dominican Republic [18] coinciding with a *C. auris* bloodstream infection in a human patient in the same geographic area, represents a particularly compelling illustration of the One Health relevance of marine mammal candidiasis. In human healthcare, *C. auris* has emerged as a major global threat due to its multidrug resistance, environmental persistence, and ability to cause nosocomial outbreaks [75]. The detection of this same pathogen in a marine mammal raises critical questions regarding the directionality of transmission (human-to-animal, animal-to-human, or shared environmental source) and suggests that marine facilities may serve as unrecognized reservoirs or sentinel sites for emerging resistant pathogens. Whole genome sequencing assigned the dolphin isolate to clade I (closely related to Indian strains), which is the most geographically widespread clade of *C. auris* globally [18]. This finding underscores the urgency of integrating marine mammal fungal surveillance into broader human public health surveillance frameworks.

Practical implementation of One Health surveillance in marine mammal facilities should include: (1) routine antifungal susceptibility screening of both symptomatic and asymptomatic animals, as recommended by Biancani et al. [19]; (2) standardized diagnostic and culture protocols, such as the in-house respiratory fungal culture protocol proposed by Marques et al. for bottlenose dolphins [41]; (3) environmental sampling of pool water and facility surfaces to monitor for resistant *Candida* strains [19]; (4) coordination between veterinary and human medical microbiology laboratories to enable genotypic comparison of isolates across species; and (5) monitoring of wastewater and coastal water quality for the presence of antifungal residues and resistant fungal strains [88]. Such integrated surveillance would enable early detection of emerging resistance patterns and zoonotic or reverse zoonotic transmission events, informing timely public health responses.

### 7.4. Conservation Implications

Endangered marine mammals are also at risk of fungal infections such as candidiasis [12]. Between 1955 and 2018, 14% of marine mammal species have been victims of infectious disease-induced mass deaths and 40% of those are endangered or vulnerable to extinction [89]. Hawaiian monk seals are vulnerable to morbillivirus outbreaks that would cause disastrous impact on their small population of about 1400 individuals [90], vaquitas, the critically endangered with fewer than 19 individuals remaining [91] and North Atlantic right whales, for which an unusual mortality event has persisted since 2017 [92], and whose limited and dispersed populations result in disease outbreaks that would have devastating effects [92]. Even minimal rates of mortality caused by opportunistic agents like *Candida* species can have huge effects on population viability and recovery patterns of small, isolated population [93]. Effective conservation strategies will require knowledge of epidemiology, risk factors, and management of candidiasis and other infectious diseases [69,94].

## 8. Future Directions and Research Needs

Significant knowledge gaps remain regarding candidiasis in marine mammals, which opens up opportunities to conduct research in the future and better comprehend, prevent, and manage such infections [3,7,12].

### 8.1. Epidemiological Surveillance

Comprehensive surveillance programs are required to define the actual incidence and the geographic location of candidiasis in the captive and wild marine mammal populations [3,12]. Standardized diagnostic protocols would allow making a comparison between facilities and regions [7,41]. The patterns of resistance, new species, and time change could be noted under the impact of the long-term monitoring programs [68]. Special focus must be put on the endangered populations with schemes of conservation focusing on infectious diseases threats [12].

Marques et al. accentuated that targeted fungal diagnostic testing on managed cetaceans is normally conducted after the development of clinical symptoms, which can hamper achievement of clinical results [41]. The authors recommended the adoption of regular fungal surveillance and screening measures as an intervention strategy in preventive medicine due to the growing problem of fungal disease and the occurrence of pathogen resistance. Their own protocol proved that point-of-care diagnostic testing can offer early and valid results, especially useful in the areas that do not have special reference laboratories of mycology. With the impacts of climate change and fungus adaptative thermotolerance taking shape very fast, surveillance protocols have to be continually changed to deal with the emerging fungal pathogens [41].

### 8.2. Pharmacology and Therapeutics

There are significant knowledge gaps in the antifungal pharmacokinetics and pharmacodynamics of marine mammals [95,96]. The species-specific studies are necessary to determine the best dosage regime, possible drug interactions, and tissue distribution and excretion routes [7,96,97]. Resistant infections could have an option with the investigation of new therapeutic methods, such as combination therapies, immunomodulators, and alternative antifungal agents [10,19,98]. Evidence-based medicine in the healthcare of marine mammals would be reinforced through clinical trials that evaluate the efficacy and safety of treatment [3].

### 8.3. Immunology and Pathogenesis

The hosts of *Candida* infections in marine mammals demonstrate a poorly defined immune response [10,63,99]. Innate and adaptive immune mechanisms, recognition of immune deficiencies that predispose to infection, and the impact of environmental stressors on immune functioning should be addressed in research [13,65,100,101]. Molecular host-pathogen interactions would be applicable in the identification of new therapeutic targets or disease biomarkers to detect the disease at its early stages [10,69].

### 8.4. Environmental and Ecological Studies

*Candida* ecology in marine environments should be investigated to learn about the transmission and persistence of diseases [10,45]. Among research questions, the environmental reservoirs, characterization of fungi communities in various marine environments, anthropogenic effects on fungal distribution, and climate change effects on fungal ecology and pattern of diseases are identified [102,103]. Research ought to be conducted on the status of water quality, water pollution, and other environmental elements in the rise of diseases [3,13,69].

### 8.5. Antifungal Resistance

Antifungal resistance in marine mammal isolates is a phenomenon that needs to be characterized by further studies of its mechanisms, epidemiology, and clinical implications [10,14,15]. The studies are recommended to define mechanisms of resistance on the molecular level, monitor resistance trends over time and across different geographic areas, examine how the environment of antifungal exposure can select resistance, and devise methods to prevent and control resistant infections [7,104]. The current problem of using antifungals in the management of marine mammals is underlined by the outbreak of azole-resistance *Candida* and *Aspergillus* isolates in captive dolphins, along with the necessity to develop antifungal susceptibility testing and stewardship programs [14,97].

### 8.6. One Health Approaches

Interdisciplinary One Health approaches should investigate connections between human, animal, and environmental health [3,84]. Assessment of zoonotic and reverse zoonotic risk transmission, the use of marine mammals as environmental health sentinels, exploring shared environmental exposures to resistant organisms, and integrated surveillance and response systems are some of the areas of priority [45,82]. Collaboration among human physicians, veterinarians, environmental scientists, and public health professionals is essential [69,105].

### 8.7. Prevention Strategies

Evidence based prevention methods, which would be developed and validated, would help minimize disease occurrence in captive populations, and may benefit wild animals [3,105]. The studies should assess the desirable water quality parameters of marine mammal facilities [97,106], efficacy of various disinfection measures [7,107] stress prevention in imprisonment and rehabilitation facilities. [108,109,110] and the building of quarantine procedures to new or stranded animals [111,112]. Research on possible vaccines or immunological prophylaxis is a more distant goal [63,69].

### 8.8. Diagnostic Innovation

Clinical management of fungal infections in the marine mammal would be improved through development of rapid, sensitive and species-specific diagnostic tests [19,41]. Point-of-care diagnostics using blow samples, non-invasive sampling methods, and biomarkers for early disease detection deserve investigation [41,113,114]. Improvement of serological methods and testing of molecular diagnostic systems using marine mammal samples would promote the improvement of diagnostic capacities [76,115]. The stress biomarker such as cortisol levels of the blubber biopsies are a promising tool that can be used to assess the health condition of free-ranging cetaceans [116].

## 9. Conclusions

This review highlights the growing importance of *Candida* infections as an emerging health issue in cetaceans and pinnipeds. A number of major conclusions can be inferred out of this synthesis of the available literature. First, although *Candida albicans* is still the most commonly isolated species in marine mammals, the rise of non-*albicans* microorganisms such as *C. tropicalis*, *C. parapsilosis*, *Nakaseomyces glabratus*, and most prominently, the multi-drug-resistant *Candida auris* in captive dolphins, is a notable shift observed in specific facilities that requires enhanced surveillance and adaptation of treatment regimens. Second, antifungal resistance data from marine mammal isolates, including reports of up to 100% azole resistance in individual facilities (based on limited sample sizes from single aquaria in Japan and Italy using CLSI M27 methodology), represent concerning facility-level findings that may reflect broader patterns of antimicrobial resistance across species, although multi-center studies are needed to confirm whether these represent global trends. The observation that resistant strains have been isolated from animals with no documented antifungal exposure suggests that aquatic environments may serve as reservoirs for resistance determinants, though indirect environmental selection through agricultural azole runoff cannot be excluded. Third, there are notable anatomical predilections in clinical manifestations observed in cetaceans and pinnipeds: cetaceans are highly vulnerable to respiratory and systemic infections through the blowhole whereas pinnipeds have a high prevalence of mucocutaneous disease at body orifices. The huge mortality rate in acute systemic candidiasis, despite intensive antifungal therapy, highlights the significance of early diagnosis and prevention measures. Fourth, the One Health paradigm provides a vital framework for understanding candidiasis in marine mammals. The reported cross-species infections of the same genotypes between dolphins and their caregivers, in addition to the indication of environmental contamination by human-associated *Candida* species, highlight the bidirectional nature of pathogenic exchange and the zoonotic potential of these infections.

Lastly, the knowledge gaps remain quite significant, including lack of species-specific pharmacokinetic information on antifungal agent use, lack of established clinical breakpoints on marine mammal isolates, as well as incomplete knowledge concerning the environmental factors that promote antifungal resistance development in marine ecosystem. The future research priorities must include standardized diagnostic guidelines, pharmacological investigations in target species, longitudinal monitoring plans and the analysis of the new therapeutic modalities such as combination therapy and new antifungal agents. To sum up, *Candida* infections in marine mammals should not be viewed solely as a veterinary issue, but also as a sentinel indicators of wider environmental and population health-related concerns.

To cope with this new scourge, interdisciplinary collaboration over the long term is necessary in terms of marine mammal medicine, medical mycology, environmental science, and community health within a comprehensive One Health approach.

## Figures and Tables

**Table 1:** *Candida* species documented in marine mammals.

*Candida* Species	Group	Host Species	Clinical Presentation	Isolation Origin	Captive/Wild	Antifungal Resistance	Reference
Clinically Significant and/or Drug-Resistant Species							
*C. albicans*							
	Cetaceans	*Tursiops truncatus* (common bottlenose dolphin), *Tursiops aduncus* (Indo-Pacific bottlenose dolphin), *T. aduncus* × *T. truncatus* hybrids, *Delphinapterus leucas* (beluga whale), *Globicephala melas* (long-finned pilot whale), *Phocoena phocoena* (harbor porpoise), *Lagenorhynchus* obscurus (dusky dolphin), *Lagenorhynchus* acutus (Atlantic white-sided dolphin), *Lagenorrhynchus obliquidens* (Pacific white-sided dolphin), *Pseudorca crassidens* (false killer whale), *Steno bredanensis* (rough-toothed dolphin), *Orcinus orca* (killer whale), *Grampus griseus* (Risso’s dolphin)	Asymptomatic colonization to severe systemic disease: respiratory infection (blowhole/pulmonary), keratomycosis, chronic cutaneous candidiasis, oral thrush with oral ulcers, gastric/esophageal distress, intestinal candidiasis, anorexia, reluctance to swallow, vomiting, head shaking, retching, lethargy, erratic behavior, abnormal respiratory exhalation, malodorous breath, sputum discharge, fever, skin discoloration (darkened areas and/or white patches), abnormal feces (grey/mucoid/stringy), death in disseminated cases	Blowhole, cornea, oral cavity, tongue, esophagus, gastric fluid/content, feces, anus, skin	Both	Azole resistance reported, susceptible: AMB, MCF, no echinocandin resistance reported	[8,11,15,19,21,23,24,25]
	Pinnipeds ^†^	*Phoca vitulina* (harbor seal), *Halichoerus grypus* (grey seal), *Callorhinus ursinus* (northern fur seal), *Zalophus californianus* (California sea lion), *Mirounga angustirostris* (northern elephant seal)	Nasal discharge, lip inflammation, periocular alopecia, vaginitis, dermatitis	Nares, lips, eye, flipper, anus, vagina, tail	Captive	Candidiasis treated successfully with ketoconazole, no MIC data reported	[9,26]
*C. tropicalis*							
	Cetaceans	*T. truncatus*, *T. aduncus* × *T. truncatus* hybrids, *P. crassidens*, *L.oscurus*, *Kogia sima* (dwarf sperm whale)	Systemic candidiasis with digestive symptoms, cutaneous lesions, oral thrush with oral ulcers, respiratory colonization/infection of blowhole, asymptomatic carriage	Blowhole, feces, gastric fluid/content, oral cavity, tongue, skin	Both	High azole resistance reported, susceptible: AMB, no echinocandin resistance reported	[14,19,21,25,27]
*C. parapsilosis*							
	Cetaceans	*Tursiops* truncatus, *L. obscurus*, *Balaena mysticetus* (bowhead whale)	Colonization and infection of skin and mucosa (lesional and non-lesional skin), gastrointestinal and respiratory colonization/infection, asymptomatic carriage, systemic candidiasis reported in mixed infections with other *Candida* spp.	Blowhole, feces, gastric fluid, oral cavity, skin	Both	Azole resistance reported, susceptible to echinocandins	[19,21,24,27,28]
*Nakaseomyces glabratus* *(anamorph:* *C. glabrata)*							
	Cetaceans	*T. truncatus*	Asymptomatic colonization of upper gastrointestinal and respiratory tracts, chronic cervical abscess described in a bottlenose dolphin (neck soft-tissue infection)	Gastric content, blowhole, oral cavity, feces, cervical abscess tissue	Both	Azole resistance reported	[19,29,30]
*C. auris*							
	Cetaceans	*T. truncatus*	Colonization	Pharyngeal	Captive	Fluconazole-resistant, susceptible to echinocandins	[18]
*Pichia kudriavzevii (anamorph:* *C. krusei)*							
	Cetaceans	*Lagenorhynchusobliquidens*, *L. obscurus*, *B. mysticetus*	Fatal intestinal candidiasis (fibrinosuppurative enteritis), oral/upper digestive candidiasis with skin lesions, colonization (skin, lesional and non-lesional)	Intestinal tissue, blowhole, stomach fluid, anus, skin (biopsies and swabs)	Both	Intrinsically azole- resistant	[24,28,31]
*C. zeylanoides*							
	Cetaceans	*Eubalaena australis* (southern right whale)	Infection	Skin	Wild	Not reported	[32]
*Yarrowia lipolytica (anamorph:* *C. lipolytica)*							
	Cetaceans	*B. mysticetus*	Colonization (skin, lesional and non-lesional)	Skin	Wild	Not reported	[28]
	^†^ Pinnipeds	*H. grypus*, *P. vitulina*	Fungal dermatitis (proliferative, alopecic, erythematous, thickened, nonpruritic skin lesions)	Trunk, face (muzzle, periorbital region), flippers (nail bed area)	Captive	Not reported	[33]
Colonization-Only Species							
*Meyerozyma guilliermondii (anamorph: C.* *guilliermondii)*							
	Cetaceans	*D. leucas*, *T. truncatus*, *B. mysticetus*	Colonization (skin, lesional only)	Blowhole, Fecal/anus, skin	Both	Not reported	[20,21,28]
*Debaryomyces hansenii* *(anamorph:* *C. famata)*							
	Cetaceans	*T. truncatus*, *D. leucas*, *B. mysticetus*	Colonization (skin, lesional and non-lesional)	Fecal/anus, blowhole, gastric, feces, skin	Both	Not reported	[20,21,28]
*C. ciferrii*							
	Cetaceans	*D. leucas*	Gastrointestinal colonization	Anus	Captive	Not reported	[20]
*Diutina rugosa (anamorph:* *C. rugosa)*							
	Cetaceans	*T. truncatus*, *B. mysticetus*	Colonization (skin, lesional and non-lesional)	Blowhole, gastric fluid, feces, skin	Both	Not reported	[21,28,29]
*C. lambica*							
	Cetaceans	*D. leucas*	Colonization	Blowhole	Captive	Not reported	[20]
*Kluyveromyces marxianus (anamorph:* *C.pseudotropicalis)*							
	Cetaceans	*D. leucas*	Gastrointestinal colonization	Anus	Captive	Not reported	[20]
*C. stellatoidea*							
	Cetaceans	*B. mysticetus*	Colonization (skin, lesional and non-lesional)	Skin	Wild	Not reported	[28]
*Pichia inconspicua (anamorph: C. inconspicua*/*Torulopsis inconspicua)*							
	Cetaceans	*B. mysticetus*	Colonization (skin, lesional and non-lesional)	Skin	Wild	Not reported	[28]
*C. intermedia*	Cetaceans	*B. mysticetus*	Colonization (skin, lesional and non-lesional)	Skin	Wild	Not reported	[28]
*C. viswanathii*							
	Cetaceans	*B. mysticetus*	Colonization (lesional skin only)	Skin	Wild	Not reported	[28]
*C. utilis*							
	Cetaceans	*B. mysticetus*	Colonization (skin, lesional and non-lesional)	Skin	Wild	Not reported	[28]
*Torulopsis candida*							
	Cetaceans	*D. leucas*, *T. truncatus*, *B. mysticetus*	Colonization	Blowhole, anus, gastric, feces, skin	Both	Not reported	[20,28,29]
*Clavispora lusitaniae (anamorph:* *C. lusitaniae)*							
	Cetaceans	*T. truncatus*	Colonization	Anus, blowhole	Wild	Not reported	[21]
*C. humicola*							
	Cetaceans	*B. mysticetus*	Colonization (skin, lesional and non-lesional)	Skin	Wild	Not reported	[28]

^†^ To date, *C. albicans* and *Y. lipolytica (formerly C. lipolytica)* are the only *Candida* species documented to cause clinical disease in captive pinnipeds [9,20,33]. AMB = amphotericin B; MCF = micafungin; MIC = minimum inhibitory concentration; R = resistant. Taxonomic note: current nomenclature is used throughout this table. Species listed under their teleomorph names include: *Nakaseomyces glabratus* (anamorph: *Candida glabrata*), *Pichia kudriavzevii* (anamorph: *C. krusei*), Meyerozyma *guilliermondii* (anamorph: *C. guilliermondii*), *Debaryomyces hansenii* (anamorph: *C. famata*), *Diutina rugosa* (anamorph: *C. rugosa*), *Kluyveromyces marxianus* (anamorph: *C. pseudotropicalis*), Pichia inconspicua (anamorph: *C. inconspicua*), *Clavispora lusitaniae* (anamorph: *C. lusitaniae*), and *Yarrowia lipolytica* (anamorph: *C. lipolytica*). Species are grouped by clinical significance: clinically significant and/or drug-resistant species (documented infections or antifungal resistance) and colonization-only species (isolated without documented disease).

**Table 2:** Clinical manifestations of candidiasis by anatomical system.

Clinical Manifestation	Cetaceans—Infected Sites	Pinnipeds—Infected Sites
Respiratory Candidiasis	Blowhole, lungs [7,8] ^a^	Nares (nasal discharge) [9] ^b^
Oral and Oropharyngeal	Tongue, buccal mucosa, pharynx, esophagus [8] ^a^	Commissures of the mouth [9] ^b^
Cutaneous Candidiasis	Body orifices: genital slit, anus [8,11] ^b^	Mucocutaneous junctions: commissures, periocular areas, nares, anus, vagina, flippers (nail beds); white plaques, alopecia, ulceration with purulent discharge [9,33] ^a^
Gastrointestinal	Esophagus, stomach, intestines [8,31] ^a^	GI tract involvement may occur but has not been documented as specific case reports; a rise in gastrointestinal candidiasis has been reported in marine mammals, including pinnipeds [7,10] ^c^
Ocular Candidiasis	Cornea (keratomycosis) [23] ^c^	Periocular only: alopecia, inflammation around the eyes [9] ^c^
Systemic/Disseminated	Lungs, heart, kidney, liver, spleen, lymph nodes, brain [8] ^b^	Not well documented for *Candida* species [7] ^c^

^a^ Common: reported across multiple studies and/or species; ^b^ occasional: documented in several cases; ^c^ rare: limited to single case reports.

**Table 3:** Documented antifungal resistance in cetacean *Candida* isolates. Documented antifungal resistance in pinniped candida isolates.

Species	Resistance Pattern	Host/Location	Year	Reference
*C. albicans*	FLC-R, ITC-R, VRC-R; Susceptible: AMB, MCF	Bottlenose dolphins, Japan	2022	[15]
*C. albicans*, *C. tropicalis*, *N. glabratus*, *C. parapsilosis*	100% azole resistance; Susceptible: echinocandins CAS, AND, MCF)	Bottlenose dolphins, Italy	2025	[19]
*C. tropicalis*	ITC-R, VRC-R (2/3 isolates)	Bottlenose dolphins, Japan	2019	[14]
*C. tropicalis*	Azole-R; Shared genotypes ST232, ST933 with humans	Clinical + non-clinical sources (marine mammals + environment), Japan	2022	[76]
*N. glabratus*	Reduced azole susceptibility (intrinsic); ITC-R	Bottlenose dolphins, Italy and Japan	2019/2025	[14,19]
*C. auris*	FLC-R (MIC 32 μg/mL [Vitek]); Susceptible: echinocandins, rezafungin, ibrexafungerp, manogepix	Bottlenose dolphins, the Dominican Republic	2025	[18]
*P. kudriavzevii*	Intrinsic FLC resistance	Pacific white-sided dolphins, USA	2010	[31]
*C. albicans*, * C. tropicalis*	FLC R or SDD (86% *C. albicans*, 80% *C. tropicalis*); ITC R or SDD (80% both species)	Captive dolphins	2010	[22]
**Species**	**Resistance Pattern**	**Host/Location**	**Year**	**Reference**
*C. albicans*	Controlled with ketoconazole (5 mg/kg BID, 10 mg/kg SID); no MIC data	Gray seal, Harbor seal, California sea lion, Northern fur seal, Northern elephant seal, USA	1985	[9]
*Yarrowia lipolytica* (*formerly Candida lipolytica*)	No susceptibility data; treated with itraconazole	Gray seals, Harbor seals, USA	1992–1994	[33]

FLC = fluconazole; ITC = itraconazole; VRC = voriconazole; AMB = amphotericin B; MCF = micafungin; R = resistant; SDD = susceptible dose-dependent; MIC = minimum inhibitory concentration.

## Data Availability

The original contributions presented in this study are included in the article. Further inquiries can be directed to the corresponding authors.
